# The dark side of avocados: a review of anthracnose and stem-end rot in postharvest fruit

**DOI:** 10.3389/fmicb.2025.1644061

**Published:** 2025-10-10

**Authors:** Valentina Valencia Bernal, Nathanial J. Boeckman, Srđan G. Aćimović, Fatemeh Khodadadi

**Affiliations:** ^1^Department of Microbiology and Plant Pathology, University of California, Riverside, Riverside, CA, United States; ^2^Plant Pathology Laboratory, School of Plant and Environmental Sciences, Alson H. Smith Jr. Agricultural Research and Extension Center, Virginia Polytechnic Institute and State University, Winchester, VA, United States

**Keywords:** avocado, postharvest diseases, stem-end rot, anthracnose, body rot

## Abstract

Avocados are a key global fruit crop with rising international demand. However, postharvest diseases like anthracnose and stem-end rot (SER) can lead to significant economic losses, with incidence rates surpassing 30% in some regions. The latent nature of these infections complicates detection and management, affecting fruit quality and marketability. This review examines the pathogens behind these diseases, highlights advancements in detection technologies such as the use of biochemical and non-destructive methods and explores host-pathogen interactions through emerging omics approaches. We also evaluate the impact of preharvest practices on disease outcomes and current management strategies, including the growing potential of biological control agents, systemic resistance inducers, and natural product-based formulations as sustainable tools that complement synthetic fungicides. Finally, we highlight implications for fruit quality and consumer perception, along with critical research gaps, particularly the imbalance between anthracnose and SER studies in avocados, and outline future directions for improving postharvest disease control in avocados.

## 1 Introduction

The avocado (*Persea americana* Mill.), a key player in the global agricultural economy, was valued at $15.83 billion in 2023, projected to reach $26.04 billion by 2030 (Avocado Market Size and Outlook, 2023). Mexico leads production with contributions from Colombia, the Dominican Republic, and Peru (FAO, 2024). In the U.S., “Hass” avocados dominate, comprising 95% of the market with per capita consumption averaging 8.43 pounds annually (World Population Review, 2025). The cultivar’s success stems from its hybrid origins, combining the Mexican race’s cold tolerance and early maturity with the Guatemalan race’s thick skin for durability and marketability (Schaffer et al., 2013; Hass, 1935).

Preserving avocado marketability depends on maintaining postharvest quality. However, postharvest diseases such as anthracnose (body rot), and stem-end rot (SER) caused by fungi from the *Colletotrichum* species, and Botryosphaeriaceae family, respectively, cause significant losses (Everett, 2020; Fuentes-Aragón et al., 2018; Korsten, 1997; Rivera et al., 2017; Sonavane and Venkataravanappa, 2022). These pathogens often remain latent, activating during ripening and storage to cause decay, reduce shelf life, and increase commercial rejection rates. For example, Colombian packinghouses reported rejection rates up to 70.1% for SER and 52.5% for anthracnose, leading to substantial economic losses (Ramírez-Gil et al., 2020). Similar losses, up to 40% in Kenya and 60% in untreated Mexican fruit, underscore the severity of these fungal diseases (Shivachi et al., 2023; Herrera-González, 2017). In contrast, comparable data are not currently available for the United States, highlighting a gap in postharvest disease monitoring efforts. Despite advancements, effective and sustainable control remains challenging, emphasizing the need for continued research into pathogen biology and innovative control strategies (Herrera-González et al., 2021).

This review comprehensively examines the biology and distribution of anthracnose and SER pathogens; highlights advance in their detection and explores their interactions with the avocado host via omics approaches. We assess how pre-harvest conditions influence disease outcomes and evaluate current and emerging management strategies. Finally, we consider these infections’ broader implications on fruit quality and consumer perception. By consolidating this research, this review aims to identify key knowledge gaps in avocado postharvest diseases and guide future efforts.

## 2 Postharvest diseases: anthracnose and stem-end rot

Anthracnose, caused by species in the genus *Colletotrichum*, and stem-end rot primarily affiliated to fungi within Botryosphaeriaceae family (Akgül et al., 2016; Giblin et al., 2018; Guarnaccia et al., 2016; Twizeyimana et al., 2013a; Valencia et al., 2019), represent major postharvest challenges for avocado production worldwide (Armand and Jayawardena, 2024; Fischer et al., 2019). While both diseases often manifest during fruit ripening and storage due to latent infections established in the field, they exhibit distinct characteristics in terms of causal agents, infection sites, and symptom development. Members of the *C. gloeosporioides* complex have also been implicated in SER (Everett, 2020; Everett et al., 2018; Galsurker et al., 2018).

**Figure 1:**
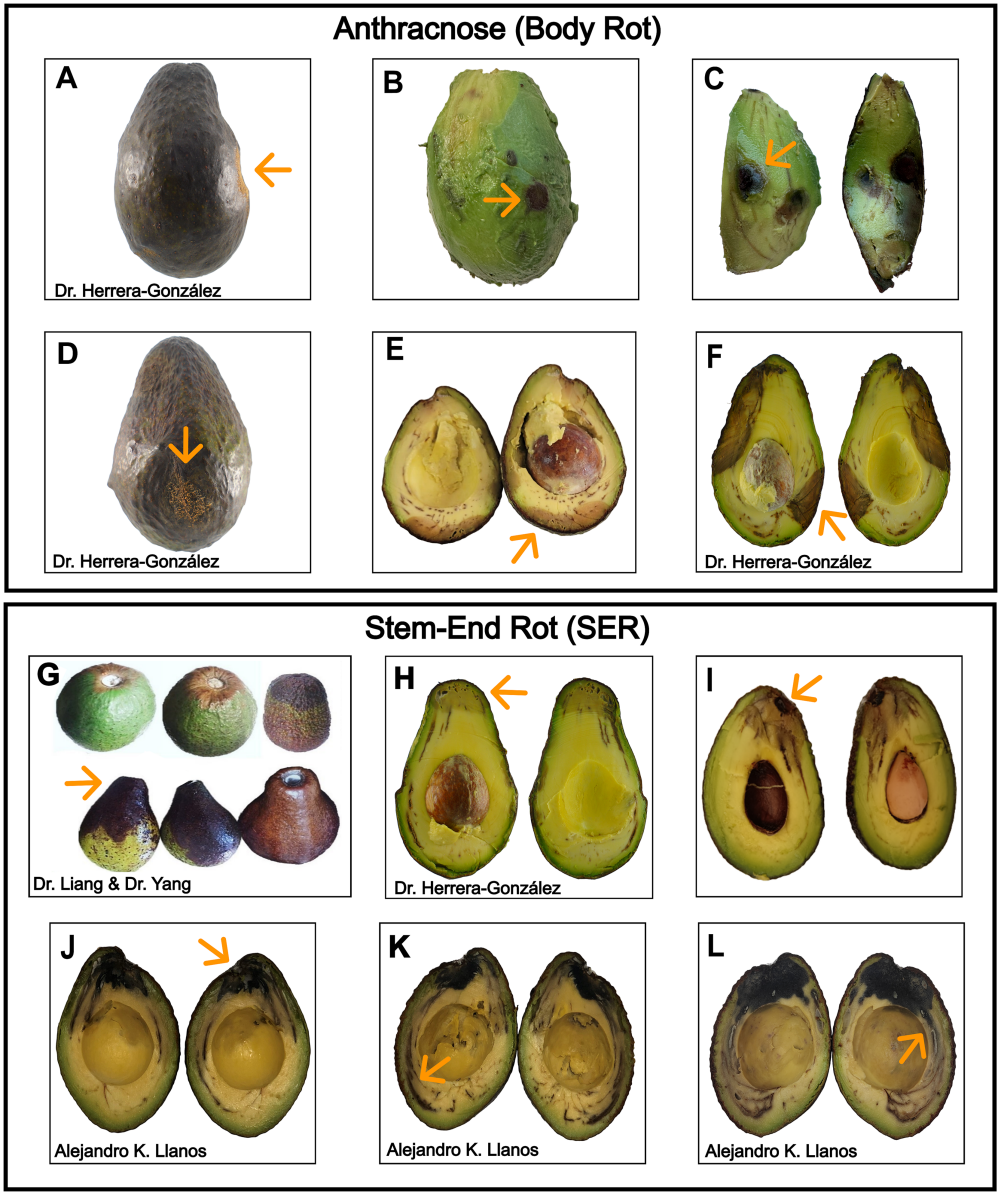
Symptoms of anthracnose and stem-end rot on avocado fruit. **(A–F)** Body rot symptoms include sunken lesions **(A,D)**, internal decay **(B,C)**, and cross-sectional views showing lesion development **(E,F)**. **(G–L)** SER symptoms include discoloration and darkening at the stem-end **(G)**, with internal browning and vascular invasion visible **(H–J)**. Infected vascular strands appear dark brown to black, forming an advancing boundary between healthy and diseased tissue **(K,L)**.

*Colletotrichum* species follow a hemibiotrophic infection cycle, colonizing fruit asymptomatically until ripening triggers a necrotrophic phase (Siddiqui and Ali, 2014). Anthracnose lesions appear as sunken (Figures 1A,D), dark (Figures 1B,C), water-soaked areas that progressively enlarge on the body of the avocado fruit (Figures 1E,F), often becoming covered with pink to orange spore masses under humid conditions (Siddiqui and Ali, 2014). The disease affects avocados both pre- and postharvest, damaging leaves and causing body rot in fruit during storage and market ripening (Dann et al., 2013; Everett and Pak, 2002; Everett, 2020; Freeman et al., 1998). In contrast, in preharvest stages, Botryosphaeriaceae fungi are associated with avocado branch canker, manifesting as sunken, resinous lesions on branches and potentially leading to dieback (Eskalen and McDonald, 2009; Twizeyimana et al., 2013a; Valencia Bernal et al., 2025). Postharvest, SER is initiated at the stem attachment site, with spores reaching the fruit via rain splash, pruning wounds, or insect activity, establishing latent infections (Eskalen et al., 2013). Affected fruits initially exhibit surface discoloration (Figure 1G), shriveling around the stem base, and softening (Karunanayake and Adikaram, 2020; Möller et al., 2025). In some cases, fungal mycelium becomes visible at the stem scar. Advanced SER leads to water-soaked lesions, tissue breakdown, and a sharp internal boundary between healthy and decayed zones (Figures 1H–L) (Fostvedt et al., 2024).

### 2.1 Taxonomy and distribution

The genus *Colletotrichum* includes over 250 species grouped into 15 species complexes (Talhinhas and Baroncelli, 2021), distinguished by molecular sequence analyses and morphological aspects (Cannon et al., 2012; Damm et al., 2010, 2012a, 2012b, 2013, 2019). In avocados, anthracnose is primarily associated with the *C. gloeosporioides* (CGSC) *C. acutatum* (CASC), and *C. boninense* (CBSC) species complex (Fischer et al., 2019; Fischer and Firmino, 2023; Fuentes-Aragón et al., 2020; Giblin et al., 2018; Iñiguez-Moreno et al., 2021). Over a dozen *Colletotrichum* species have been confirmed as causal agents of anthracnose in avocados across multiple countries, including Israel, Kenya, Mexico, Australia, Ghana, and Brazil. These species include *C. acutatum, C. aenigma, C. alienum, C. fioriniae, C. fructicola, C. gigasporum, C. gloeosporioides, C. godetiae, C. karstii, C. nupharicola, C. perseae, C. siamense*, and *C. theobromicola* (Dann et al., 2013; Hernández-Lauzardo et al., 2015; Kimaru et al., 2018; Sharma et al., 2017; Velázquez-del Valle et al., 2016). Regional surveys highlight variability in species prevalence shaped by environmental conditions (Dowling et al., 2020; Fuentes-Aragón et al., 2018; Kwon et al., 2020; Silva-Rojas and Ávila-Quezada, 2011; Soares et al., 2021). Thermal preferences of *Colletotrichum* species complexes (CGSC optimal 26–27°C, tolerant to 38–40°C) (Salotti et al., 2022); CASC optimal 20–22°C (Khodadadi et al., 2023); CBSC intermediate ~25°C (CABI, 2022; Moriwaki et al., 2003) influence their geographic distribution.

The taxonomy of Botryosphaeriaceae has undergone significant revisions (Burgess et al., 2019; Phillips et al., 2013; Slippers et al., 2017), with genus-level classification advanced through morphological and molecular analyses (Liu et al., 2012; Phillips et al., 2019; Slippers et al., 2004). Genera consistently associated with SER include *Botryosphaeria*, *Lasiodiplodia*, *Diplodia*, *Neofusicoccum*, and *Dothiorella* (Fostvedt et al., 2024; Galsurker et al., 2018). *Lasiodiplodia theobromae* is widely reported (Fischer et al., 2019; Llanos and Apaza, 2021; Ochoa and Vázquez, 2006; Ramírez-Gil et al., 2020; Rosado et al., 2016; Valencia et al., 2019; Wanjiku et al., 2020). Other important species include *N. luteum* (Everett, 2020; McDonald et al., 2009; Twizeyimana et al., 2013a), *Diplodia seriata* and *D. pseudoseriata* (Valencia et al., 2019), *N. parvum*, *N. mediterraneum*, *N. australe, D. mutila*, and *L. citricola* (Aćimović et al., 2018; Chen et al., 2014; McDonald et al., 2009; Moral et al., 2019).

The increasing global incidence of postharvest diseases in avocados and the identification of new pathogenic species underscores the complexity and expanding geographic distribution of species affecting avocado production. Understanding their disease cycle, infection strategies, and detection methods is crucial for developing effective disease management approaches as new species emerge across various regions.

### 2.2 Disease cycle

The disease cycles of both *Colletotrichum* and Botryosphaeriaceae responsible for these postharvest rots involve latent infections activated during fruit ripening (Fischer et al., 2019; Prusky et al., 2013). Initial infection by *Colletotrichum* spp. begins with germ tubes that develop appressoria to penetrate the fruit (Perfect et al., 1999). This is followed by a biotrophic phase until ripening triggers necrotrophy, with secretion of cell wall degrading enzymes (CWDEs) leading to sunken lesions and secondary conidia formation (Everett et al., 2008; Hartill, 1991; Hartill and Everett, 2002; Prusky et al., 2013). *Colletotrichum* overwinters as conidium or sclerotia in plant debris (Casela and Frederiksen, 1993; Stensvand et al., 2017; Yoshida et al., 2007), with infection beginning at lenticels, wounds, or compromised skin by splash-dispersed conidia germinating under wet conditions and temperatures typically ranging from 10°C to 35°C (Estrada et al., 2000; Everett and Pak, 2002; Everett et al., 2018; Morse and Faber, 2017; Salotti et al., 2022).

**Figure 2:**
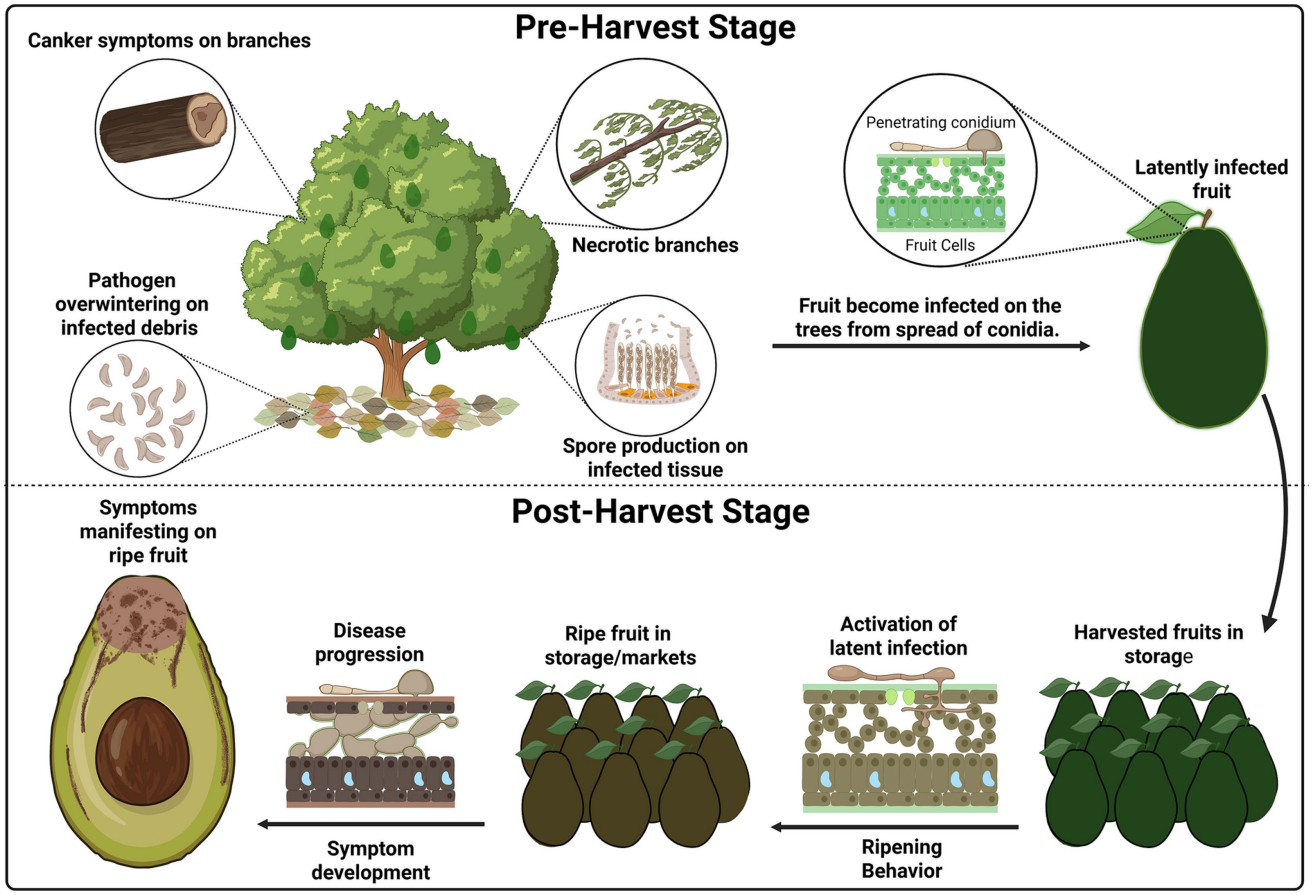
Disease cycle of stem-end rot in avocado. Pathogens survive in cankers and infected debris, producing conidia that infect fruit preharvest and establish latent infections. Symptoms develop postharvest during ripening and storage. Created in BioRender. Valencia Bernal, V. (2025) https://BioRender.com/bxrcbhq.

Botryosphaeriaceae persist in plant tissues as mycelium, pycnidia, or conidia (Galsurker et al., 2018; Johnson et al., 1992), entering the tree preharvest through natural openings or wounds, with spores dispersed by rain, wind, and insects (Eskalen et al., 2013; Hartill and Everett, 2002; Karunanayake and Adikaram, 2020; Navarro et al., 2022). Inside the fruit, a quiescent biotrophic state transitions to necrotrophy during ripening due to biochemical changes weakening host defenses (Alam et al., 2020; Galsurker et al., 2018; Prusky and Lichter, 2007), exacerbated by high humidity (>90%) and warm temperatures of 20–25°C (Bill et al., 2014; Defilippi et al., 2015). During necrotrophy, CWDEs are also secreted (Defilippi et al., 2018; Nagel et al., 2021a), leading to internal decay from the stem end through the vascular system (Fostvedt et al., 2024; Möller et al., 2025) (Figure 2). Pycnidia forms in necrotic tissue, releasing secondary conidia that serve as inoculum for future infections (Twizeyimana et al., 2013a). The persistence of Botryosphaeriaceae fungi in dead and living branches, infected fruit, and even soil ensures that the disease cycle continues year after year, making early and efficient disease management essential (Llanos and Apaza, 2021).

## 3 Detection and diagnosis

Accurate detection of *Colletotrichum* and Botryosphaeriaceae species is essential for managing postharvest diseases and preserving avocado fruit quality (Alam et al., 2020; Fischer et al., 2019; Karunanayake and Adikaram, 2020). Traditional visual inspection is unreliable due to the pathogens’ quiescent nature (Matsui et al., 2023). This limitation is problematic in “Hass” avocados, where the dark, textured skin obscures early lesion development more than in lighter cultivars such as ‘Fuerte’ and ‘Bacon’ (Bill et al., 2014; Fischer et al., 2019). As a result, undetected infections continue to cause economic losses throughout the supply chain (Ramírez-Gil et al., 2020, 2021).

Various detection methods for Botryosphaeriaceae- and *Colletotrichum*-induced diseases have also been tested across diverse crops such as mango, kiwifruit, blueberries, citrus, strawberries, olives, apples, and peppers, providing valuable insights that could be integrated into avocado disease monitoring systems (Cabrera Ardila et al., 2020; Clark et al., 2004; Fazari et al., 2021; Jeong et al., 2024; Li et al., 2021; Wang et al., 2021; Wu et al., 2023; Xu et al., 2016; Yang et al., 2022). Furthermore, with the pathogens’ similar infection strategies, there is strong potential to adapt or co-develop methods for dual diagnosis (Fischer et al., 2019).

### 3.1 Molecular detection methods

Polymerase chain reaction (PCR) is a widely used method for detecting *Colletotrichum* and Botryosphaeriaceae species. However, due to the genetic similarities, single-locus PCR often lacks the resolution for species-level identification. Multilocus phylogenetic analysis has become standard for resolving closely related taxa, using combinations of housekeeping genes (Table 1) (Phillips et al., 2013, 2019; Vieira et al., 2020).

**Table 1:** PCR primers used in species delimitation of *Colletotrichum* and Botryosphaeriaceae fungi.

Product name	Gene	Primer	Sequence (5′–3′)	Length (bp)	Reference
Internal Transcribed Spacer^ab^	ITS	ITS1- F	CTTGGTCATTTAGAGGAAGTAA	290	White et al. (1990)
		ITS4	TCCTCCGCTTATTGATATGC	330	
β-tubulin^a^	Bt2	BT2-A	GGTAACCAAATCGGTGCTGCTTTC	495	Glass and Donaldson (1995)
		BT2-B	ACCCTCAGTGTAGTGACCCTTGGC		
β-tubulin 2^b^	TUB2	T1	AACATGCGTGAGATTGTAAGT	1,500	O’Donnell and Cigelnik (1997)
		T2	TAGTGACCCTTGGCCCAGTTG	730	
Elongation factor-1α^ab^	TEF-1α	EF1-728F	CATCGAGAAGTTCGAGAAGG	350	Carbone and Kohn (1999)
		EF1-986R	TACTTGAAGGAACCCTTACC		
Calmodulin^b^	CAL	CL1C	GAATTCAAGGAGGCCTTCTC	756	Weir et al. (2012)
		CL2C	CTTCTGCATCATGAGGTGGAC		
Glyceraldehyde-3-phosphate dehydrogenase^b^	GAPDH	GDF-F	GCCGTCAACGACCCCTTCATTGA	270	Templeton et al. (1992)
		GDF-R	GGGTGGAGTCGTACTTGAGCATGT		
Glutamine Synthetase^b^	GS	GSF	ATGGCCGAGTACATCTGG	900	Stephenson et al. (1997)
		GSR	GAACCGTCGAAGTTCCAC		
Actin^b^	ACT	Act512F	ATGTGCAAGGCCGGTTTCGC	300	Carbone and Kohn (1999)
		Act783R	TACGAGTCCTTCTGGCCCAT		
DNA Lyase^b^	APN2	ColDL-F3	GGGAGAAGCGAACATACCA	756	Rojas et al. (2010)
		CgDL-R1	GCCCGACGAGCAGAGGACGTAGTC		
Intergenic spacer and partial mating type (Mat1-2) gene^b^	ApMat	CgDL-F6	AGTGGAGGTGCGGGACGTT	870	Rojas et al. (2010)
		CgMAT1F2	TGATGTATCCCGACTACCG		

a Primer set used for Botryosphaeriaceae identification.

b Primer set used for *Colletotrichum* spp. identification.

ab Primer set used for both pathogens.

For anthracnose, PCR targeting the internal transcribed spacer (ITS) and β-tubulin (TUB2) regions enables genus-level identification (Chen et al., 2006; Vieira et al., 2020). To distinguish species within *Colletotrichum*, additional loci such as glutamine synthetase (*GS*), glyceraldehyde-3-phosphate dehydrogenase (*GAPDH*), calmodulin (*CAL*), actin (*ACT*), chitin synthase (*CHS-1*), DNA lyase (*APN2*), mating-type protein (*MAT1-2*) and L-arabinitol dehydrogenase (ladA) (Khodadadi et al., 2020; McHenry and Aćimović, 2024) are often employed. Specific combinations enhance resolution for species complexes (Khodadadi et al., 2020; Talhinhas and Baroncelli, 2021). For example, the CGSC is best resolved using APN2/MAT-IGS and GAP2-IGS, while CASC species are more accurately distinguished with HIS3, GAPDH, and TUB2 (Khodadadi et al., 2020).

For Botryosphaeriaceae, commonly sequenced loci include ITS, TUB2, and elongation factor-1α (TEF-1α) (Phillips et al., 2013, 2019), mirroring their role in *Colletotrichum* species differentiation. In avocados, these loci have enabled the first report of species-level detection of *Neofusicoccum australe* (Akgül et al., 2016), *Botryosphaeria dothidea* (Qiu et al., 2020), and *Diaporthe rudis* (Torres et al., 2016). These loci continue to be the standard for species identification within the Botryosphaeriaceae family (Avenot et al., 2023; Valencia Bernal et al., 2025).

Beyond avocado, cross-crop assays offer insight into anthracnose and SER detection. Xu et al. (2016) developed species-specific PCR primers targeting the ITS and TUB2 gene regions, facilitating accurate species identification of *Lasiodiplodia theobromae, Botryosphaeria dothidea*, and *Neofusicoccum parvum* found in stem blight samples (Xu et al., 2016). Similarly, Ni et al. (2012) developed a nested multiplex PCR (mPCR) assay for mangoes, enabling simultaneous detection of *L. theobromae*, *N. parvum*, *N. mangiferae*, and *Fusicoccum aesculi,* at fungal DNA concentrations as low as 100 femtograms to 1 picogram (Ni et al., 2012). In soybeans, Chen et al. (2006) developed a multiplex PCR assay using ITS1/ITS2-based primers to detect *C. gloeosporioides* and *C. truncatum*, with amplification at 100 nanograms and no cross-reactivity with non-target DNA, highlighting its specificity and sensitivity (Chen et al., 2006).

Quantitative PCR (qPCR) enhances conventional PCR by providing real-time pathogen DNA detection, crucial for identifying latent infections and supporting early surveillance during both orchard and postharvest storage due to its high sensitivity; qPCR has been instrumental in distinguishing closely related *Colletotrichum* species within the *C. gloeosporioides* and *C. acutatum* complexes (Cao et al., 2023; Garrido et al., 2009). Recent multilocus qPCR assays, combining markers such as ITS, GAPDH, and TUB2, have successfully resolved species-level identities at femtogram DNA concentrations. In apples, bitter rot pathogens were distinguished with a detection limit of 0.5 pg (McHenry and Aćimović, 2024), while ITS-based quantification of *C. fructicola* in asymptomatic *Camellia* tissue demonstrated robust sensitivity and specificity (Cao et al., 2023). Similar strategies have proven effective for tracking colonization and resistance responses in infected tissues (Yang et al., 2022), and for identifying quiescent infections in strawberry transplants using both SYBR Green and TaqMan chemistries (Rahman et al., 2019). In olives, assays targeting *C. acutatum* allowed detection as early as 16 h after inoculation, and an alternative virulence-gene assay (klap1) achieved femtogram-level detection (10.14 fg per reaction) (Azevedo-Nogueira et al., 2021). One of the more innovative developments, a spore-based qPCR for peppers, enabled *Colletotrichum* identification from a single conidium without requiring DNA extraction, greatly streamlining diagnostic workflow while maintaining accuracy (Jeong et al., 2024). This extraction-free model holds strong promise for anthracnose detection in avocado, particularly in high-throughput or time-sensitive settings.

qPCR advancements have improved SER detection with duplex and triplex TaqMan assays enabling simultaneous detection of *N. parvum*, *B. dothidea*, and broader Botryosphaeriaceae groups in various plants and environmental samples (Romero-Cuadrado et al., 2023, 2024). Billones-Baaijens et al. (2018) developed a multispecies qPCR assay targeting the β-tubulin gene to quantify Botryosphaeriaceae inoculum in vineyards. This robust assay detected 10 species with high specificity and sensitivity, proving valuable for diagnostics, spore dispersal modeling, and orchard sanitation (Billones-Baaijens et al., 2018).

The high sensitivity and specificity of PCR and qPCR-based assays make them indispensable tools for early detection and disease monitoring, especially in avocado-exporting regions where preventing latent infections is critical for marketability. While these remain as cornerstones for pathogen detection in laboratory settings, their reliance on specialized equipment has prompted researchers to explore alternative technologies that offer greater accessibility and field applicability.

Loop-mediated isothermal amplification (LAMP) has emerged as a powerful alternative to conventional PCR and qPCR, for rapid, low-cost and on-site detection of plant pathogens, including those responsible for anthracnose and SER. Operating at a constant temperature (60–65°C), LAMP eliminates the need for thermal cyclers, making it highly suited for in-field diagnostics in orchards or export settings (Law et al., 2015). Although LAMP is not yet widely used for postharvest avocado pathogens, research on other avocado and fruit crop diseases demonstrates its considerable promise (Kamali Dashtarzhaneh et al., 2025).

In mango, a calmodulin-targeted LAMP assay reliably identified members of the *Colletotrichum gloeosporioides* species complex at DNA concentrations ranging from 0.1 to 10 ng/mL (Hattori et al., 2021). Lan et al. (2020) pushed this sensitivity even further, detecting *C. gloeosporioides* in guava at just 10 femtograms of DNA, roughly a thousand times more sensitive than standard PCR and confirming its utility for detecting latent infections in asymptomatic fruit (Lan et al., 2020). In soybean, a LAMP assay for *C. truncatum* detected as little as 100 pg/μL and successfully identified the pathogen in field samples and seed lots containing only 10 conidia per 50 g (Tian et al., 2017). More recently, efforts have improved not just sensitivity, but usability. Cui et al. (2024) developed two assays for *C. siamense*, one using cresol red for a visible color shift, the other incorporating a fluorescent TaqMan probe for real-time readout. Both reached a detection limit of 50 copies/μL and successfully identified soil inoculum at 10^4^ CFU/g (Cui et al., 2024). In strawberries, Wu et al. (2019) demonstrated how LAMP could be adapted to monitor fungicide resistance. Their assay simultaneously detected *C. gloeosporioides* and its G143A resistance mutation with just 10 pg of DNA, and by pairing the assay with a lateral flow device, they completed field detection within an hour, no lab required (Wu et al., 2019).

While most existing assays target anthracnose pathogens, efforts to adapt the technology for SER pathogens are growing. Wang et al. (2021) developed an assay for *Botryosphaeria dothidea* in kiwifruit that matched qPCR in sensitivity, reaching 10 picograms. In avocado, Madhu et al. (2025) created a tef1-α-based assay for *Lasiodiplodia pseudotheobromae*, capable of distinguishing it from closely related species and detecting infections down to 25 pg of DNA. King et al. (2024) took it a step further with a genus-level *Lasiodiplodia* assay, validated not only in culture but also in drone-captured air samples, detecting as little as 6.25 pg of DNA and demonstrating LAMP’s potential for environmental monitoring.

However, LAMP does come with its setbacks and limitations. Primer design is technically demanding, requiring 4–6 primers that must bind with high precision to 6–8 distinct target sites. Even minor mismatches can result in non-specific amplification or false positives, particularly under field conditions with inconsistent sample quality (Rolando et al., 2020). Visual detection formats, like color changes in dye-based assays, can be difficult to interpret under inconsistent lighting or with colored or cloudy plant extracts (Garg et al., 2022). Ultimately, while LAMP offers considerable benefits in speed and portability, its successful application hinges on rigorous assay validation, appropriate user training, and careful interpretation of results (Wong et al., 2018).

### 3.2 Biochemical and non-destructive detection methods

Advancements in biochemical and non-invasive sensor technologies have introduced powerful tools for early detection of postharvest diseases in avocado, complementing PCR-based diagnostics with real-time, tissue-preserving options (Zamir et al., 2020). Platforms based on metabolomics, volatile profiling, spectroscopy, and artificial intelligence (AI) imaging are increasingly capable of detecting latent infections before visible symptoms appear (Cabrera Ardila et al., 2020; Chauhan et al., 2024; Jansen et al., 2011; Khlaif et al., 2024).

Metabolomic and volatile compound profiling show promise as An et al. (2024) used ultra-high performance liquid chromatography-mass spectrometry (UHPLC–MS) and gas chromatography–mass spectrometry (GC–MS), to identify early-stage biomarkers, including shikimate, succinic acid, and tyrosine, in *L. theobromae*-infected grapefruit 1 day post-inoculation, predating visible symptoms (An et al., 2024). Similarly, Moalemiyan et al. (2006) used GC–MS to identify volatile organic compounds (VOCs) in mangoes infected with *L. theobromae* and *C. gloeosporioides*, classifying SER with 1-pentanol and ethyl boronate, and anthracnose with thujol. Their discriminant model achieved up to 100% classification accuracy (Moalemiyan et al., 2006). In papaya, Tan et al. (2024) used solid-phase microextraction and GC–MS analysis to identify microbial fermentation markers (e.g., acetic acid) and healthy fruit biomarkers such as methyl butanoate and benzyl isothiocyanate, providing a model for avocado disease screening (Tan et al., 2024).

Isothermal microcalorimetry (IMC) has also emerged as a non-destructive biochemical tool capable of monitoring real-time fungal metabolic activity through heat flow. Betancourt-Rodríguez et al. (2023) standardized IMC conditions to characterize *Colletotrichum* species isolated from various fruits, including avocados, by analyzing thermokinetic parameters. Using the total heat (H_t_) and maximum growth rate (μ_max_), as well as multivariate analyses, they demonstrate consistent and reproducible heat signatures across the isolates. This revealed physiological distinctions that could support future detection efforts (Betancourt-Rodríguez et al., 2023). Building on this, Betancourt-Rodríguez et al. (2025) applied canonical discriminant analysis to group fungal genera based on thermokinetic profiles. Furthermore, they trained a neural network model using the resulting canonical variables. The model achieved high accuracy (95%) in distinguishing *Colletotrichum*, *Penicillium*, and *Alternaria* genera, highlighting IMC’s potential as a rapid and non-invasive diagnostic method (Betancourt-Rodríguez et al., 2025). Spectroscopy and near-infrared (NIR) imaging offer non-destructive diagnostic potential. NIR spectroscopy accurately classified bruising in “Hass” avocados (over 85% accuracy within 1–2 h) (Wedding et al., 2012). Fourier transform NIR spectroscopy (FT-NIRS) assessed anthracnose-induced rot in avocados with 65–84% accuracy (Wedding et al., 2019). Visible-NIR spectroscopy also significantly reduced chilling injury rated in kiwifruit (Clark et al., 2004). These optical technologies demonstrate robust capabilities for early and non-invasive detection of both physical damage and latent disease in horticultural crops.

AI-integrated imaging offers even higher-resolution diagnostics. In Japan, Matsui et al. (2023) used deep learning segmentation (U-net++) on X-ray images to classify rot-affected avocado tissue at the pixel level with 98% accuracy and a root mean square error of just 3.15% in rot area quantification. For surface-based diagnostics, Valiente et al. (2021) applied convolutional neural networks (CNNs) to identify anthracnose lesions with 92% accuracy based on texture and color pattern. Campos-Ferreira et al. (2023) used machine learning classifiers, including Random Forest (RF) and multilayer perceptron (MLP), to distinguish healthy, anthracnose-, and scab-infected fruit. RF and MLP models achieved 98% overall classification accuracy and an F1 score of 98% for anthracnose detection.

Even though many of these tools were developed for anthracnose or in other crops, particularly those detecting VOCs, metabolic shifts, or structural changes, they offer a compelling framework for broader avocado application. Given the latent infection strategies shared by anthracnose and SER, these non-destructive technologies can serve as screening or early-warning systems, enhancing disease control, reducing postharvest losses, and complementing molecular assays.

## 4 Plant-pathogen interactions: the use of omics

### 4.1 Metabolomics

Metabolomics has provided key insights into the biochemical changes that occur during avocado ripening and infection. Through high-resolution analytical techniques such as UHPLC–MS, and other variations of metabolic work, researchers have identified metabolites involved in defense, stress signaling, and disease progression (Castro-Moretti et al., 2020). One of the most consistent findings is the decline of antifungal compounds, such as epicatechin, chlorogenic acid, and other flavonoids, as the fruit ripens (Di Stefano et al., 2017; López-Cobo et al., 2016; Ramos-Aguilar et al., 2021; Younis et al., 2022). This reduction, observed across peel, pulp, and seed tissues, weakens internal defenses and correlates with the activation of latent infections by pathogens like *C. gloeosporioides* and *N. parvum* (Ardi et al., 1998; Bowen et al., 2018; Prusky et al., 1989, 2013). Phenolic content also varies by cultivar and growing region. Avocados from Spain, Chile, Peru, and Australia show significant differences in flavonol glycosides and phenolic acids (Lyu et al., 2023; Serrano-García et al., 2023), and peel tissues consistently retain higher flavonoid levels than pulp. Rapid phenolic degradation in the pulp may help explain why internal tissues are often the first to succumb to fungal attack (Lyu et al., 2023; Younis et al., 2022).

Pathogens also contribute to disease through their own metabolite production. *N. parvum* secretes virulence factors like hydroxymellein, isosclerone, and tyrosol, which induce necrosis and cellular damage (Abou-Mansour et al., 2015; Evidente et al., 2010). Other *Neofusicoccum* species produce fatty acid-derived toxins such as linoleic and azelaic acid, which disrupt host oxidative balance and promote tissue colonization (Salvatore et al., 2018). *C. gloeosporioides* expresses laccases that degrade polyphenols like epicatechin, undermining antifungal defenses and enhancing lipoxygenase-mediated cell wall breakdown (Guetsky et al., 2005; Bill et al., 2017). Additionally, Botryosphaeriaceae species interfere with jasmonic acid and salicylic acid signaling, suppressing host immune activation (Abou-Mansour et al., 2015).

These metabolic disruptions collectively reduce host resistance and accelerate decay. Yet, metabolomics alone does not reveal the regulatory pathways underlying these shifts. To address this, transcriptomics has emerged as a powerful tool for examining gene expression changes associated with infection, ripening, and postharvest stress (Zhang et al., 2022; Wang and Huo, 2022).

### 4.2 Transcriptomics

Transcriptomics has significantly advanced our understanding of the molecular responses of avocado during ripening, pathogen attack, and postharvest storage. These insights have revealed critical shifts in plant defense signaling, oxidative stress responses, and fungal virulence regulation.

Foundational studies generated *de novo* transcriptome assemblies for avocado, mango, and macadamia (Chabikwa et al., 2020), enabling high-resolution analyses of tissue-specific gene expression. In Mexican avocado, transcript profiling identified regulators of fatty acid metabolism and ripening across different tissues and developmental stages (Ibarra-Laclette et al., 2015). Subsequent studies using 1-methylcyclopropene (1-MCP) demonstrated that ethylene inhibition alters expression of key genes involved in ripening and long-term storage responses (Olivares et al., 2022).

Pathogen-focused transcriptomic studies have uncovered immune activation pathways. In fruit infected with *C. gloeosporioides* and treated with chitosan, Xoca-Orozco et al. (2017) identified upregulation of phenylpropanoid biosynthesis and hormone signaling, suggesting chitosan functions as an elicitor of avocado defense responses. In roots infected with *Fusarium kuroshium*, genome-wide expression profiling revealed coordinated upregulation of genes and microRNAs related to hormone pathways, secondary metabolism, and cell wall modification, highlighting systemic defense reprogramming (Pale et al., 2024). Storage-associated transcriptomic changes have also been linked to fruit decay. Cold storage conditions modulate antioxidant systems by altering expression of phenolic biosynthesis genes and enzymes such as superoxide dismutase (SOD) and peroxidase (POD), both crucial for managing oxidative stress during shelf life (Chirinos et al., 2023). Together, these findings underscore the utility of transcriptomics in unraveling avocado responses to developmental, environmental, and biotic stress cues.

Other pathogen-focused transcriptomic studies have revealed critical virulence strategies employed by both *Colletotrichum* spp. and Botryosphaeriaceae during crop infection. Transcriptomic studies have revealed that *Colletotrichum* spp. coordinate distinct gene expression programs across infection stages to support their hemibiotrophic lifestyle (O’Connell et al., 2012). Early infection involves the upregulation of genes linked to appressorium formation, host penetration, and stress tolerance, including melanin biosynthesis enzymes, fatty acid metabolism genes, and redox regulators (O’Connell et al., 2012; Liang et al., 2018). During biotrophy, small secreted proteins (SSPs), LysM effectors, and other immune-suppressing factors are expressed to evade host detection, while the transition to necrotrophy is marked by broad induction of carbohydrate-active enzymes, proteases, tannases, and necrosis-inducing proteins (Liang et al., 2018; Ozbudak et al., 2025). Comparative studies show that dicot-infecting species exhibit greater transcriptional plasticity and deploy a more diverse repertoire of effectors and degradative enzymes than monocot specialists, reflecting host-driven genomic and regulatory adaptation (Baroncelli et al., 2024).

On the host side, transcriptomic studies of resistant cultivars of walnut and strawberry have shown that *Colletotrichum* infection activates multiple layers of defense, including early expression of pattern recognition receptors (*FLS2*, *EFR*), calcium-dependent protein kinases (CDPKs), and pathogenesis-related genes such as *PR1* (Fang et al., 2021; Wang et al., 2017). In addition, effector-triggered immunity is activated through resistance genes including *RIN4*, *RPM1*, and *RPS2*, with downstream signaling involving WRKY transcription factors, respiratory burst oxidase homologs (*Rboh*), and calcium channels (CNGCs) (Fang et al., 2021; Wang et al., 2017). These transcriptional changes reflect a dynamic defense strategy tailored to both the biotrophic and necrotrophic phases of infection.

Transcriptomic analyses have similarly revealed that Botryosphaeriaceae pathogens deploy a diverse array of virulence mechanisms during host colonization. In grapevines infected with *Lasiodiplodia theobromae*, Zhang et al. (2019) reported strong activation of genes involved in plant hormone signaling, immune receptor recognition, and defense-related secondary metabolism. Both pathogen-associated molecular pattern (PAMP)-triggered and effector-triggered immunity pathways were engaged, with increased expression of pattern recognition receptors, such as FLS2 and CERK1, MAP kinase signaling components (MPK3/6), WRKY transcription factors, and resistance (R) genes, including *RPM1* and *RPS2*. Expression of phenylpropanoid pathway genes and reactive oxygen species (ROS)-associated enzymes further reflected the activation of both local and systemic defense mechanisms in the host (Zhang et al., 2019). Other transcriptomic studies have identified additional fungal virulence factors. *L. theobromae* upregulates genes involved in phenolic catabolism and cell wall degradation under heat stress conditions, including salicylate hydroxylase, pectate lyase, and catechol dioxygenases (Paolinelli-Alfonso et al., 2016). Moreover, the secreted endopolygalacturonase LtEPG1 in *L. theobromae* has been shown to induce host cell death and activate defense-related transcription when transiently expressed in *Nicotiana benthamiana*, suggesting a dual role as both a virulence factor and elicitor (Thilini Chethana et al., 2020; Peng et al., 2025). Together, these findings suggest that Botryosphaeriaceae pathogens utilize a combination of enzymatic degradation, detoxification, and immunomodulatory strategies to facilitate latent colonization and postharvest symptom expression.

### 4.3 Multi-omics

**Figure 3:**
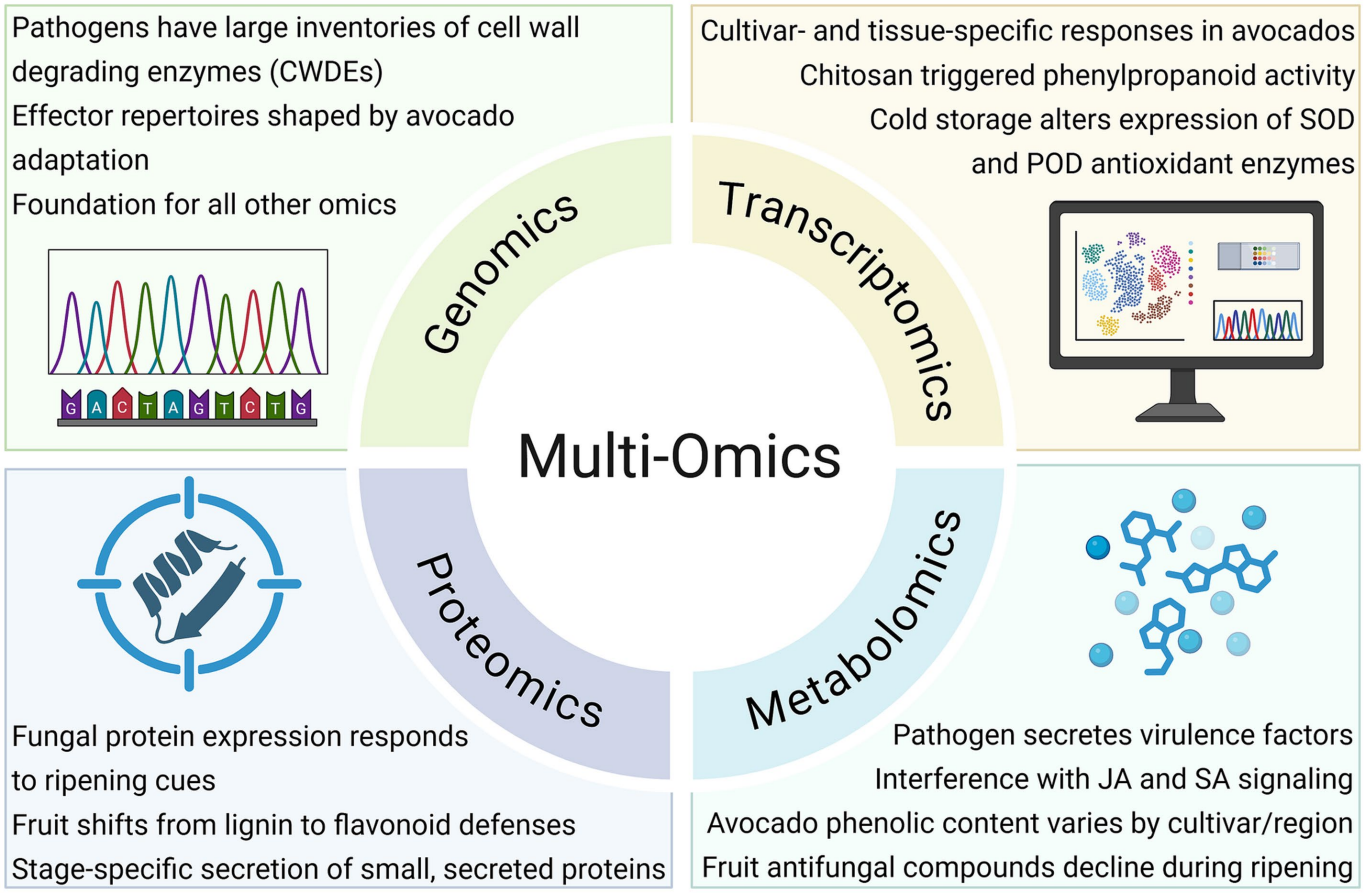
Multi-omics insights into host-pathogen interactions during postharvest disease. Genomics, transcriptomics, proteomics, and metabolomics can collectively reveal pathogen virulence factors, infection strategies, host defense responses, and metabolic shifts, providing an integrated framework for early detection and disease management. (JA = Jasmonic Acid; SA = Salicylic Acid; SOD = Superoxide dismutase; POD = Peroxidase) Created in BioRender. Valencia Bernal, V. (2025) https://BioRender.com/u28ig2w.

Multi-omics approaches have enabled researchers to unravel the complex molecular networks regulating postharvest disease development in avocado. While metabolomics has revealed the depletion of antifungal compounds during ripening and transcriptomics has uncovered gene-level immune responses, combining these with genomics and proteomics offers a more integrated view of host-pathogen interactions (Figure 3). This systems-level perspective is critical for understanding how pathogens exploit ripening-associated vulnerabilities to initiate infection and accelerate fruit decay (Núñez-Lillo et al., 2023). However, the effectiveness of multi-omics depends heavily on foundational resources. Without well-annotated genomes and reference datasets for avocado and its pathogens, interpreting gene function, expression patterns, and metabolite shifts remains challenging (Hayes et al., 2024). Nevertheless, recent work is demonstrating the power of integration.

Multi-omics approaches have begun to reveal how postharvest fungal pathogens coordinate their infection strategies in response to host physiology, particularly during avocado ripening and storage. Rather than acting through isolated molecular processes, pathogens such as *L. theobromae*, *N. parvum*, and *B. dothidea* deploy tightly regulated networks of virulence genes, identified through genomic and transcriptomic studies, that remain latent in asymptomatic fruit until triggered by postharvest stress (Nagel et al., 2021b). These networks include large inventories of CWDEs, secreted proteases, and biosynthetic gene clusters, which are transcriptionally upregulated in response to host-derived cues such as ripening-related metabolic shifts (Yan et al., 2018).

Integrating genomics and transcriptomics has shown that Botryosphaeriaceae pathogens maintain compact, repeat-poor genomes that lack the “two-speed” structure seen in many fast-evolving fungi (Nagel et al., 2021a). Yet within this stable genome context, virulence genes are co-regulated through dynamic expression modules that respond to changes in host carbohydrate availability and oxidative balance. Gene family expansions linked to stress tolerance and colonization, such as those encoding glucanases, major facilitator superfamily (MFS) transporters, and orsellinic acid biosynthesis enzymes, underscore the group’s metabolic flexibility (Liang et al., 2024). These adaptations allow pathogens to shift quickly from a quiescent state to aggressive colonization without requiring large-scale genomic reorganization.

*Colletotrichum* species, by contrast, exhibit a stage-specific infection strategy that has been elucidated through coordinated genomic, transcriptomic, and metabolomic analyses. Comparative genomics of *C. orbiculare* and *C. gloeosporioides* has revealed enriched clusters of small secreted proteins, carbohydrate-active enzymes, and effectors that support both stealth colonization and host degradation (Gan et al., 2013, 2021). Transcriptomic studies show that during biotrophic establishment, genes encoding LysM-domain proteins and immune-suppressing effectors are upregulated to evade host defenses. As infection transitions to the necrotrophic phase, CWDEs, proteases, and pectinases dominate the transcriptome, reflecting the pathogen’s shift toward tissue breakdown and nutrient acquisition (Gan et al., 2013).

Multi-omics integration has also illuminated the host response. In *Stylosanthes* plants infected with *Colletotrichum*, Jiang et al. (2021) demonstrated that infection triggers upregulation of flavonoid biosynthesis genes, including phenylalanine ammonia-lyase and flavonoid 3′-hydroxylase, resulting in elevated accumulation of antifungal metabolites such as kaempferol, quercetin, and naringenin. This biochemical shift occurs alongside downregulation of lignin biosynthesis genes, suggesting a strategic allocation of defense resources from structural reinforcement to metabolite-based antifungal defense (Jiang et al., 2021).

## 5 Factors influencing postharvest disease

The development and severity of postharvest diseases in avocados are shaped by a complex interplay of preharvest and postharvest factors. Orchard management practices, harvest timing, and ripening physiology directly affect fruit structure, pathogen resistance, and storage performance (Arpaia et al., 1987, 2004; Defilippi et al., 2015). Fruit quality is established during development and maturation; postharvest conditions can only preserve, not improve, this quality (Everett and Pak, 2001).

### 5.1 Orchard practices

Orchard practices significantly influence avocados’ postharvest quality, fruit firmness, ripening behavior, disease susceptibility, and storage potential (Arpaia et al., 2004; Everett and Pak, 2001). Factors such as irrigation uniformity, tree nutrition, fruit positioning, and sanitation practices directly affect fruit structural integrity, skin resilience, and metabolic stability, influencing postharvest performance and marketability (Defilippi et al., 2015).

Both drought and excessive rainfall significantly affect fruit quality and postharvest disease susceptibility. Inconsistent irrigation has been linked with lenticel damage, irregular ripening, and increased susceptibility to anthracnose and SER (Ramírez-Gil et al., 2021). Excessive rainfall before harvest raises internal water content, leading to cell breakdown during ripening and increased vulnerability to infection (Pak et al., 2003). Water-deficit stress further impacts ripening dynamics; fruits losing water rapidly (2.9% fresh weight/day) ripened 40% faster than those with slower loss (0.5% per day), due to elevated ethylene production and mesocarp softening (Adato and Gazit, 1974). Additionally, drought-stressed fruit develop higher internal temperatures, and without transpiration cooling, postharvest heat buildup exacerbates physiological disorders. Brief exposure to temperatures >25°C for 24 h significantly increases SER and anthracnose incidence (Kassim et al., 2013; Arpaia et al., 2018).

Calcium (Ca) deficiencies increase susceptibility to SER and anthracnose by weakening cell wall structure and accelerating ripening (Thorp et al., 1997; Ramírez-Gil et al., 2021). Calcium crosslinks pectin, limiting enzymatic degradation, nutrient leakage, and microcrack formation that facilitate fungal colonization (Kirkby and Pilbeam, 1984; Messenger et al., 1997; Tingwa and Young, 1974). While foliar Ca (NO_3_)_2_ applications have shown mixed results, some improving firmness and reducing ethylene production (Barrientos-Priego et al., 2016), root uptake is generally more effective (Partridge et al., 2002; Messenger et al., 1997). Late-harvested fruit typically contain less calcium, correlating with higher incidence of vascular browning and fungal infection (Thorp et al., 1997; Chaplin and Scott, 1980). Other nutrients also influence disease risk: excess nitrogen promotes rapid ripening and softening, while higher potassium supports skin strength and even ripening (Penter and Stassen, 2000; Kirkby and Pilbeam, 1984).

Orchard canopy structure influences light penetration, humidity, and airflow, all of which affect postharvest disease risk. Fruit from the lower canopy which is exposed to higher humidity and reduced airflow, exhibits increased incidence of anthracnose and SER (Defilippi et al., 2015; Everett and Pak, 2001). Dense canopies also support higher fungal inoculum loads, raising infection rates at harvest (Everett et al., 2007). Canopy position also affects fruit maturity and dry matter (DM) content; lower-canopy and early-season fruit typically accumulate less DM and are more susceptible to diseases (Shezi et al., 2020).

Preharvest fungal inoculum levels in orchards are a major predictor of postharvest rot incidence. The amount of infected leaf tissue within an orchard has been directly correlated with the level of postharvest rots in fruit harvested. Quantification of spore load from leaf surfaces, dead branches, and stem tissue, revealed that these act as major reservoirs for *Colletotrichum* spp. and *Botryosphaeria* spp. (Everett et al., 2003). Pruning strategies that reduce canopy density and improve sunlight penetration have been shown to lower postharvest disease rates, as fruit from open canopies exhibit thicker skin, better calcium accumulation, and reduced moisture retention (Ramírez-Gil et al., 2021). These physiological conditions, set during the preharvest period, ultimately influence how fruit responds to harvest and ripening.

### 5.2 Harvest timing and ripening behavior

Harvest timing significantly impacts postharvest disease resistance. Fruit harvested at peak physiological maturity generally exhibits greater resilience to fungal infection than overripe or immature fruit (Arpaia et al., 2018). Overripe fruit’s increased lipids and ethylene accelerate softening, promoting pathogen invasion. Immature fruit ripens unevenly, developing defects that predispose it to decay (Arpaia et al., 2018; White et al., 2009). This vulnerability is further shaped by the fruit’s postharvest ripening behavior. Unlike many fruits, avocados remain physiologically mature but unripe on the tree due to suppressed ethylene biosynthesis (Hershkovitz et al., 2009; Kende, 1993). Once harvested, they enter climacteric ripening, marked by increased ethylene production and respiration (Lelièvre et al., 1997). These ripening changes weaken fruit defenses: mesocarp softening can cause cuticle microcracks, enabling pathogen entry (Everett, 2020; Everett et al., 2008), while levels of antifungal compounds such as dienes and phenolic acids decline (Prusky et al., 1989, 2013; Prusky and Lichter, 2007). Environmental and orchard level conditions further influence ripening behavior. Warmer preharvest conditions, such as higher mean minimum air temperatures and cumulative degree-days, accelerate postharvest softening and peel color change (Rivera et al., 2017). In contrast, fruit from trees with higher leaf area index and canopy density ripen more slowly, likely due to altered carbohydrate allocation and photosynthetic activity (Rivera et al., 2017). From orchard conditions to harvest timing and postharvest ripening, each stage shapes the fruit’s physiological trajectory and its susceptibility to disease.

## 6 Management strategies

Effective postharvest avocado disease management requires both preharvest risk mitigation and postharvest handling. Integrated Disease Management (IDM) combines cultural, chemical, biological, and physical controls for sustainable disease reduction (Pandey et al., 2016; Gurr, 2021). However, IDM adoption is limited in avocado due to challenges such as lacking predictive models; nevertheless, its principles are vital for cutting chemical dependence and extending control efficacy (He et al., 2016).

### 6.1 Cultural practices

Cultural strategies aim to reduce inoculum sources and minimize fruit exposure to conditions that favor pathogen infection. These practices represent targeted interventions used in orchards and packing systems to limit disease development before and during harvest.

Regular removal of fallen fruit, dead wood, and mummified tissues disrupts the disease cycle and limits fungal inoculum in the orchard (Everett et al., 2018; Galsurker et al., 2018). Maintaining an open canopy through selective pruning improves airflow and reduces humidity, two conditions that suppress spore germination and fungal colonization. Pruning should be performed with sanitized tools to prevent mechanical spread of pathogens within and between trees (Everett, 2020; Fostvedt et al., 2024).

Clean harvest cuts are essential for minimizing infection risk. Using clippers instead of pulling fruit prevents stem tearing, a primary entry point for SER pathogens (Mazhar et al., 2018; Koo-Lee, 2024). Harvesting during or immediately after rainfall should be avoided to reduce surface moisture that favors fungal germination. Postharvest, fruit should be cooled to 4°C within 6 h to suppress ripening and pathogen growth, while avoiding lower temperatures to prevent chilling injury. Maintaining cold chain continuity and minimizing handling delays are critical for preserving fruit quality and reducing disease expression during storage and distribution (Morse and Faber, 2017).

### 6.2 Chemical control

Fungicide applications remain a key component of preharvest disease management in avocado, particularly for controlling anthracnose. In the U.S., only azoxystrobin and copper-based fungicides are registered for use against anthracnose preharvest, while no fungicides are currently approved for managing SER (Morse and Faber, 2017). Postharvest fungicide applications are not standard practice in the U.S., where emphasis remains on cultural practices (Morse and Faber, 2017).

Fungicides trials against Botryosphaeriaceae fungi, remain experimental and are unregistered for commercial avocado production (Valencia Bernal et al., 2025). In Southern California, field trials showed that preharvest applications of azoxystrobin combined with propiconazole significantly protected against *Neofusicoccum* spp. (Twizeyimana et al., 2013b). New Zealand trials explored copper alternatives for preharvest fungicides. Pyraclostrobin and boscalid effectively controlled anthracnose and SER, often surpassing copper. Fluazinam matched copper for anthracnose but was inconsistent against SER (Everett et al., 2011).

Although limited in avocado, prochloraz showed strong postharvest efficacy in research. A 5-min dip in 500 ppm prochloraz significantly reduced anthracnose and SER (Danderson, 1986). Similar success occurred in mangoes when prochloraz was applied alone or with fludioxonil as a heated dip (Swart et al., 2009). However, prochloraz is not registered for postharvest use in the U.S. and is being phased out in Europe due to toxicological concerns (Shimshoni et al., 2020). As a potential alternative, fludioxonil has demonstrated comparable efficacy against SER and anthracnose. In some avocado trials, it outperformed prochloraz in controlling early-season cultivar decay with minimal residue beyond the peel (Shimshoni et al., 2020).

Fungicide resistance is a major challenge for controlling anthracnose, particularly in *Colletotrichum* species. Resistance to various fungicide classes, including quinone outside inhibitors (QoIs) is well-documented, often linked to target gene mutations like β-tubulin (Chung et al., 2006). For instance, over 90% of *C. acutatum* isolates from Florida strawberry fields resisted azoxystrobin and pyraclostrobin (Forcelini et al., 2016). Recently carbendazim-resistant *C. fructicola* and *C. siamense* populations were identified in China with no fitness penalties (Karim et al., 2024). Besides biological resistance, fungicide regulations are stringent. Since 1990s, Europe has withdrawn over 70% of previously approved active substances due to environmental and toxicological concerns (Fussell, 2016). Active ingredients such as prochloraz are being phased out due to concerns over carcinogenicity and endocrine disruption (Leskovac and Petrović, 2023). Globally, import rejections from residue are fueling demand for safer, residue-free alternatives (Chikte et al., 2024).

### 6.3 Biological control

Microbial biocontrol agents (BCA) offer promising alternatives to chemical fungicides for managing anthracnose and SER. Bacterial species such as *Pseudomonas fluorescens* and *Bacillus subtilis* produce lytic enzymes like chitinase and β-1,3-glucanase, which degrade fungal cell walls and inhibit pathogen growth. Fortnightly applications of talc-based *P. fluorescens* formulations combined with chitin have been shown to significantly delay anthracnose symptom development postharvest (Vivekananthan et al., 2004). More recently, three strains of *Bacillus thuringiensis* were evaluated against *C. gloeosporioides*, achieving *in vivo* inhibition rates above 63%. The antagonistic activity was attributed to volatile compound production, nutrient competition, and β-1,3-glucanase secretion (Magallón-Andalón et al., 2025). Yeast-based biocontrol works by competitive exclusion, where yeasts colonize fruit surfaces, outcompeting fungal pathogens for nutrients and infection sites. For instance, *Saccharomyces cerevisiae* significantly reduced anthracnose in mangoes, suggesting potential for avocado. However, it was less effective than *P. fluorescens* (Vivekananthan et al., 2004). New findings suggest that *Meyerozyma caribbica* and *M. guilliermondii* exhibit strong antagonism against *L. theobromae*, with co-cultures outperforming individual strains in mycelial inhibition (~90%) (Ayón-Macías et al., 2025). Fungal antagonists, especially *Trichoderma* species, effectively suppress *Colletotrichum* and *Neofusicoccum* pathogens via mycoparasitism, antibiosis, and induced resistance. *T. harzianum* suppressed *C. gloeosporioides* in tropical fruits (Wijeratnam et al., 2008), while *T. atroviride* inhibited *N. pseudotrichia* growth by 55% and fully controlled SER on avocado (Wanjiku et al., 2021). These biocontrol agents could be integrated into spray programs or combined with low-risk fungicides to enhance disease suppression and reduce chemical use.

However, BCAs often show inconsistent efficacy commercially due to environmental sensitivity and competition with native microbes. For instance, *Trichoderma* species may be less antagonistic in nutrient-rich soils. Many BCAs require specific temperature or humidity, and small-scale trial success often does not translate to field-scale. Introducing non-native microorganisms also carries ecological risks like invasiveness or disrupting native communities. These limitations highlight the need for more research, better formulations, and careful ecological assessment before fully integrating BCAs into large-scale postharvest disease management (Singh et al., 2020).

To address these limitations, advances in formulation have improved the stability, viability, and delivery of microbial agents under both pre- and post-harvest conditions. In one study, electrosprayed microcapsules of *Yamadazyma mexicana* applied pre- and postharvest reduced anthracnose severity by up to 96% at 25°C and up to 93% under cold storage, with complete disease suppression (100%) when both treatments were combined, without compromising fruit quality (González-Gutiérrez et al., 2024a). Building on this work, a follow-up study developed a powdered version of the bioformulation, which preserved yeast viability, maintained antifungal activity, and reduced anthracnose severity and incidence by 88.9 and 80%, respectively, during postharvest storage, while also maintaining key physicochemical quality traits of the fruit (González-Gutiérrez et al., 2024b). Similarly, solution blow-spun nanofibers made with pullulan and loaded with *Meyerozyma caribbica* completely prevented symptom development on avocado fruits as a preventive application and reduced disease severity by up to 76% under curative application in cold storage, outperforming azoxystrobin in both cases (Vázquez-González et al., 2024). These formulation strategies enhance consistency and efficacy across various storage conditions, providing a more consistent and scalable approach to postharvest disease control.

### 6.4 Systemic resistance inducers

Instead of directly targeting fungal pathogens, systemic resistance inducers (SRIs) activate host defense pathways, priming the fruit’s immune system and reducing its susceptibility to infection (Elliston et al., 1977). These compounds stimulate enzymes such as phenylalanine ammonia-lyase (PAL), chitinase, and β-1,3-glucanase, leading to increased phenolic compound accumulation and cell wall reinforcement (Munhuweyi et al., 2020).

Phosphorus acid is an efficient SRI; a 500 mg L^−1^ preharvest application boosted PAL activity and antifungal compounds, with peak disease suppression 14 days post-treatment (Bosse et al., 2013). Similarly, 1.5% chitosan sprays enhanced PAL and epicatechin in the exocarp, improving firmness and suppressing anthracnose and SER (Munhuweyi et al., 2020; Obianom et al., 2019). Submicron chitosan dispersions also increased PAL, peroxidase (PO), polyphenol oxidase (PPO), and total phenolics in dragon fruit, reducing *C. gloeosporioides* incidence and severity (Zahid et al., 2015). In avocado, chitosan-thyme oil coatings have been shown to increase phytoalexin production and resistance under warm storage conditions (Herrera-González et al., 2021).

More recently, phenylalanine (Phe) has emerged as an SRI that activates mitogen-activated protein kinase (MAPK) cascades, WRKY transcription factors, and flavonoid biosynthesis. In mango, Phe-treated fruit showed reduced oxidative stress, increased anthocyanin levels, and suppressed *Colletotrichum* growth (Patel et al., 2023). Moreover, high-degree polymerized agave fructans (HDPAF) have been shown to trigger over 5,400 differentially expressed genes in “Hass” avocado, including key components of immune perception (FLS2, WRKY33, CRK25), MAPK signaling, and phenylpropanoid and flavonoid pathways. HDPAF treatment also delayed ethylene peaks, reduced respiration, and helped preserve fruit quality during storage, highlighting its potential as a dual-function elicitor for both disease management and shelf-life extension (Cuéllar-Torres et al., 2023).

SRIs have also demonstrated efficacy against Botryosphaeriaceae pathogens. In mango, a combination of hexanal vapor and bacterial antagonist (*Pseudomonas fluorescens*) reduced SER caused by *L. theobromae*, while increasing the activity of PAL, peroxidase (PO), polyphenol oxidase (PPO), superoxide dismutase (SOD), and catalase (CAT) (Seethapathy et al., 2016). In banana, hexanal vapor (800 ppm) completely inhibited *C. gloeosporioides* and *L. theobromae in vitro* and reduced disease incidence *in vivo* by 75.2 and 80.2%, respectively. It transiently elevated PAL, PO, PPO, and glucanase, inhibited phospholipase D, and promoted cell wall thickening, enhancing resistance and delaying ripening (Dhakshinamoorthy et al., 2020). In avocado, preventive root applications of marine extracts with potassium oxide and *Trichoderma harzianum* reduced *L. theobromae* lesion development and improved root and dry matter traits, indicating systemic defense activation (Jiménez-Ariza et al., 2023). In passion fruit, eugenol reduced lesion incidence by 87% and increased activities of PAL, chitinase, β-1,3-glucanase, PO, PPO, SOD, and CAT, alongside phenolic and flavonoid accumulation (Sun et al., 2023).

### 6.5 Plant extracts and essential oils

Plant-derived volatiles and essential oils (EOs) have emerged as promising tools in postharvest disease management. Their antifungal activity stems from multiple mechanisms such as membrane disruption, metabolic interference, inhibition of germination, and in some cases, activation of host defenses (Sivakumar et al., 2021).

Thyme oil, rich in thymol and carvacrol, has shown consistent efficacy against *C. gloeosporioides*. When applied as vapor in combination with modified atmosphere packaging, it significantly reduced anthracnose severity in avocado and extended shelf life by minimizing lesion development and ripening (Sellamuthu et al., 2013). Similarly, *Lippia sidoides* oil, incorporated into a 1% carboxymethylcellulose (CMC) edible coating, reduced infection rates while maintaining firmness and sensory quality of avocados, an effect attributed to its synergistic action of thymol and carvacrol (Antonia et al., 2024).

Other plant species such as *Lippia scaberrima L. javanica*, and *Artemisia afra* have demonstrated strong antifungal effects linked to terpenoid components like R-(-)-carvone. Their oils have been successfully tested in coating applications and in fresh-cut systems, where they reduced browning, microbial load, and helped preserve antioxidant activity on avocados (Regnier et al., 2010; Adeogun et al., 2020a, 2020b). Furthermore, clove and cinnamon oils, used in fumigation or dip treatments, delayed SER for up to 7 days at 15°C, with clove oil proving most effective against L. theobromae and Diaporthe nelumbonis (Nilmini et al., 2021).

EO volatility remains a key limitation, reducing their efficacy in storage. To overcome this, edible coatings are used to regulate EO release and improve adhesion. Chitosan and CMC-based coatings incorporating oregano or moringa extracts have demonstrated improved firmness retention, antioxidant preservation, and slowed ripening in avocado (Tesfay et al., 2021; Cenobio-Galindo et al., 2019). Basil oil embedded in a beeswax matrix similarly reduced fungal development while maintaining texture (Karunanayake et al., 2020).

Encapsulation and nano structuring have further enhanced delivery. Thyme oil-loaded chitosan nanoparticles completely inhibited *C. gloeosporioides in vitro* and reduced disease incidence by up to 60% in fruit without compromising quality (Correa-Pacheco et al., 2017). In another study, EO-chitosan formulations integrated into biodegradable polymeric nets reduced anthracnose by 80%, offering a novel packaging-based solution (Correa-Pacheco et al., 2022). Modified atmosphere packaging with lemongrass EO further suppressed anthracnose severity and preserved marketable traits such as firmness, flavor, and color (Mpho et al., 2013).

Incorporating EOs into coatings and packaging systems has expanded their utility beyond surface sprays, allowing for sustained antifungal activity and improved fruit quality. Even though these treatments may not fully replace fungicides in high-pressure disease environments, they offer a compelling alternative or complement in organic systems and export markets seeking to reduce postharvest losses while meeting sustainability standards.

## 7 Sensory and consumer impact of fungal diseases

Postharvest diseases significantly compromise avocado sensory attributes, including texture, flavor, and aroma, critically impacting consumer acceptance and marketability (Ramírez-Gil et al., 2021). While external blemishes are visible, internal quality deterioration manifesting as excessive softening, uneven ripening, off-flavors, and textural collapse is more detrimental to consumer perception (Aked, 2002). Dry matter (DM) content is a key quality determinant, high DM (>26%) correlates with a desirable buttery texture and richer flavor, whereas low DM (<20%) fruit results in a watery, bland, and less appealing fruit (Giuggioli et al., 2023). Fungal infections accelerate cell wall breakdown, lipid oxidation, and enzymatic degradation, leading to premature softening and structural collapse, making fruit feel undesirable even pre-consumption (Defilippi et al., 2018; Harker et al., 2010; Ruiz-Aracil et al., 2024). The preferred ripeness stage at purchase (medium-soft 6.5 N firmness) is also disrupted by pathogens which either accelerate or delay softening, leading to increased bruising and internal necrosis that significantly reduce purchase intent (Arpaia et al., 2015).

Beyond textural changes, disease-induced biochemical shifts influence aroma, reducing consumer acceptability. Fungal infections alter aroma by producing VOCs, modifying phenolic content, and increasing lipid oxidation (Bano et al., 2023; Fernandes et al., 2024; Obenland et al., 2012). Pathogenic fungi, including *Colletotrichum* spp. and Botryosphaeriaceae spp., disrupt natural volatiles profiles by accelerating lipid membrane breakdown, leading to an incomplete transition from immature to mature aromas (Gong et al., 2022; Obenland et al., 2012; Parthasarathy et al., 2017). Healthy ripening avocados undergo a decline in grassy aldehydes (e.g., hexanal), and an increase in desirable compounds like acetaldehyde, and β-myrcene, contributing to a creamy, nutty aroma (Obenland et al., 2012). However, fungal infections alter the trajectory, often preserving grassy aldehydes and suppressing key esters, resulting in unripe, musty, or fermented odors (Gong et al., 2022). For example, *Colletotrichum* infections trigger oxidative stress and elevate enzymes like PPO, POD, and lipoxygenase (LOX), promoting lipid oxidation and rancid volatiles formation (Fernandes et al., 2024). Similar infections in mangoes and apples increase alcohols, ketones, and aldehydes, associated with unpleasant odors (Parthasarathy et al., 2017). The increase in C6/C9 aldehydes and alcohols in infected fruit is linked to LOX pathway activation contributing to off flavors (Gong et al., 2019). Given aroma’s critical role in consumer acceptability, fungal-induced volatile disruptions emphasize the need for effective postharvest disease management to preserve avocado aroma and marketability.

While postharvest fungal diseases reduce fresh avocados marketability, the impact on processed products is poorly understood. Processing typically involves removing visibly defective fruit, disinfection and blending with additives (Koo-Lee, 2024). However, infected fruit passing visual inspection can still exhibit internal breakdown, off-flavors, and altered volatile profiles that persist post-processing, particularly in minimally processed items like guacamole. Despite rising demand for avocado products, systematic research on how infections affect processed quality is lacking. Addressing this gap is critical for developing comprehensive disease management extending beyond whole-fruit appearance.

## 8 Discussion

Research on postharvest fungal diseases in avocado exhibits a notable imbalance. While significant attention has been paid to *Colletotrichum* species and anthracnose due to their economic impact and available research tools, Botryosphaeriaceae fungi (SER pathogens), including *Neofusicoccum, Lasiodiplodia, and Botryosphaeria,* remain comparatively understudied in avocado. Much of the current knowledge is derived from other hosts, limiting avocado-specific disease models and hindering a full understanding of latent infection and unique symptom expression.

Despite advancements in molecular diagnostics (e.g., qPCR, LAMP) for asymptomatic detection, their broader adoption faces challenges. High costs, specialized equipment, training needs, and lack of standardized protocols limit implementation, particularly for small-scale producers and in resource-limited regions (O’Brien and Alamar, 2025). Similarly, multi-omics approaches are nascent, but their utility is constrained by a lack of annotated genomes, cultivar-specific infection models, and foundational omics datasets for avocado and its pathogens, hindering the identification of functional interactions.

Current management strategies largely depend on cultural practices and fungicides, especially preharvest treatments for anthracnose. However, efficacy against SER is inconsistent, and concerns over fungicide resistance and environmental impact drive the need for sustainable alternatives. While fungicides will likely persist, they can be complemented by biological control agents, systemic resistance inducers, and plant-based products. The transition of biocontrol solutions from laboratory to commercial scales has encountered hurdles like inconsistent efficacy under variable conditions, formulation stability, and regulatory complexities (Peralta-Ruiz et al., 2023).

Beyond disease control, these infections severely impact sensory attributes diminishing consumer acceptance and marketability (Gamble et al., 2010) softening, discoloration, and off-flavors (Obenland et al., 2012). As the avocado industry expands, future research must integrate traditional management with emerging biotechnologies. A multidisciplinary approach, combining agronomic, molecular, and technological innovations, is essential for ensuring long-term sustainability. By adopting a systems-based approach, from preharvest optimization to innovative management, the industry can secure more effective, sustainable, and economically viable solutions to preserve avocado quality globally.

## References

[ref1] Abou-Mansour E. Débieux J.-L. Ramírez-Suero M. Bénard-Gellon M. Magnin-Robert M. Spagnolo A.. (2015). Phytotoxic metabolites from *Neofusicoccum parvum*, a pathogen of *Botryosphaeria* dieback of grapevine. Phytochemistry 115, 207–215. doi: 10.1016/j.phytochem.2015.01.012, PMID: 25747381

[ref2] Aćimović S. G. Rooney-Latham S. Albu S. Grosman D. M. Doccola J. J. (2018). Characterization and pathogenicity of Botryosphaeriaceae Fungi associated with declining urban stands of coast redwood in California. Plant Dis. 102, 1950–1957. doi: 10.1094/PDIS-02-18-0339-RE, PMID: 30110246

[ref3] Adato I. Gazit S. (1974). Water-deficit stress, ethylene production, and ripening in avocado fruits. Plant Physiol. 53, 45–46. doi: 10.1104/pp.53.1.45, PMID: 16658649 PMC541330

[ref4] Adeogun O. O. Maroyi A. Afolayan A. J. (2020a). Quality retention of fresh-cut fruits of avocado enhanced with the essential oils from aerial parts of *Lippia javanica*, incorporated with gum Arabic edible coating. Acta Hortic. 1292, 227–238. doi: 10.17660/ActaHortic.2020.1292.30

[ref5] Adeogun O. O. Maroyi A. Afolayan A. J. (2020b). Shelf-life enhancement of fresh-cut fruits of avocado treated with the essential oils from aerial parts of *Artemisia afra*, incorporated with gum Arabic edible coating. Acta Hortic. 1292, 239–250. doi: 10.17660/ActaHortic.2020.1292.31

[ref6] Aked J. (2002). “Maintaining the postharvest quality of fruits and vegetables” in Fruit and vegetable processing: Improving quality. ed. Jongeneditor W. (Cambridge, England: Woodhead Publishing Ltd), 119–149.

[ref7] Akgül D. S. Awan Q. N. Güler P. G. Önelge N. (2016). First report of anthracnose and stem end rot diseases caused by *Colletotrichum gloeosporioides* and *Neofusicoccum australe* on avocado fruits in Turkey. Plant Dis. 100:1792. doi: 10.1094/PDIS-03-16-0279-PDN

[ref8] Alam M. W. Rehman A. Malik A. U. Ahmad S. Haider M. S. Amin M.. (2020). Dynamics of stem end rot disease of mango fruit and its management. Pak. J. Agric. Sci. 57, 63–71. doi: 10.21162/PAKJAS/19.8336

[ref9] An J.-P. Li J. Rodrigues-Stuart K. Dewdney M. M. Ritenour M. A. Wang Y. (2024). Machine learning-based metabolomics analysis reveals the early biomarkers for *Diplodia* stem-end rot in grapefruit caused by *Lasiodiplodia theobromae*. Postharvest Biol. Technol. 212:112868. doi: 10.1016/j.postharvbio.2024.112868

[ref10] Antonia B. D. de Oliveira J. da Silva P. P. M. dos Mares Biazotto A. de Toledo N. M. V. da Glória E. M.. (2024). Positive effect of *Lippia sidoides* essential oil associated with carboxymethylcellulose in the control of anthracnose in avocado. Food Prod. Process. Nutr. 6:39. doi: 10.1186/s43014-023-00209-1

[ref11] Ardi R. Kobiler I. Jacoby B. Keen N. T. Prusky D. (1998). Involvement of epicatechin biosynthesis in the activation of the mechanism of resistance of avocado fruits to *Colletotrichum gloeosporioides*. Physiol. Mol. Plant Pathol. 53, 269–285. doi: 10.1006/pmpp.1998.0181

[ref12] Armand A. Jayawardena R. S. (2024). Morphomolecular identification and pathogenicity of *Colletotrichum* species associated with avocado anthracnose in northern Thailand. Plant Pathol. 73, 186–197. doi: 10.1111/ppa.13792

[ref13] Arpaia M. L. Collin S. Sievert J. Obenland D. (2015). Influence of cold storage prior to and after ripening on quality factors and sensory attributes of ‘Hass’ avocados. Postharvest Biol. Technol. 110, 149–157. doi: 10.1016/j.postharvbio.2015.07.016

[ref14] Arpaia M. L. Collin S. Sievert J. Obenland D. (2018). ‘Hass’ avocado quality as influenced by temperature and ethylene prior to and during final ripening. Postharvest Biol. Technol. 140, 76–84. doi: 10.1016/j.postharvbio.2018.02.015

[ref16] Arpaia M. Mitchell F. Katz P. Mayer G. (1987). Susceptibility of avocado fruit to mechanical damage as influenced by variety, maturity and stage of ripeness. S. Afr. Avocado Grow. Assoc. Yearb. 10, 149–151.

[ref17] Arpaia M. van Rooyen Z. Bower J. P. Hofman P. Woolf A. (2004). Grower practices will influence postharvest fruit quality, vol. 2. Quillota, Chile: Seminario Internacional De Paltos.

[ref18] Avenot H. F. Vega D. Arpaia M. L. Michailides T. J. (2023). Prevalence, identity, pathogenicity, and infection dynamics of Botryosphaeriaceae causing avocado branch canker in California. Phytopathology 113, 1034–1047. doi: 10.1094/PHYTO-11-21-0459-R, PMID: 36510362

[ref19] Avocado Market Size and Outlook. (2023). Glob. Avocado Mark. Size Outlook 2023–2030. Available online at: https://www.grandviewresearch.com/industry-analysis/fresh-avocado-market-report (Accessed April 22, 2025).

[ref20] Ayón-Macías K. D. Ragazzo-Sánchez J. A. Calderón-Santoyo M. (2025). Antagonistic effect of co-cultures involving *Meyerozyma caribbica* and *M. guilliermondii* against postharvest pathogens of jackfruit: understanding the mechanisms behind their action. Physiol. Mol. Plant Pathol. 136:102528. doi: 10.1016/j.pmpp.2024.102528

[ref21] Azevedo-Nogueira F. Gomes S. Lino A. Carvalho T. Martins-Lopes P. (2021). Real-time PCR assay for *Colletotrichum acutatum sensu stricto* quantification in olive fruit samples. Food Chem. 339:127858. doi: 10.1016/j.foodchem.2020.127858, PMID: 32829246

[ref22] Bano A. Gupta A. Prusty M. R. Kumar M. (2023). Elicitation of fruit fungi infection and its protective response to improve the postharvest quality of fruits. Stress 3, 231–255. doi: 10.3390/stresses3010018

[ref9001] Baroncelli R. Cobo-Díaz J. F. Benocci T. Peng M. Battaglia E. Haridas S.. (2024). Genome evolution and transcriptome plasticity is associated with adaptation to monocot and dicot plants in Colletotrichum fungi. GigaScience 13, 1–18. doi: 10.1093/gigascience/giae036 PMC11212070 38940768

[ref23] Barrientos-Priego A. F. Martínez-Damián M. T. Vargas-Madríz H. Lázaro-Dzul M. O. (2016). Effect of preharvest calcium spraying on ripening and chilling injury in ‘Hass’ (*Persea americana* mill.) avocado. Rev. Chapingo Ser. Hortic. 22, 145–159. doi: 10.5154/r.rchsh.2016.04.010

[ref24] Betancourt-Rodríguez J. Ragazzo-Sánchez J. A. Sandoval-Contreras T. Calderón-Santoyo M. (2025). Discriminant analysis and neural networks for the identification of phytopathogenic fungi by isothermal microcalorimetry. Thermochim. Acta 748:179993. doi: 10.1016/j.tca.2025.179993

[ref25] Betancourt-Rodríguez J. Zamora-Gasga V. M. Ragazzo-Sánchez J. A. Zapata J. A. N. Calderón-Santoyo M. (2023). A standardized method for genus colletotrichum characterization by isothermal microcalorimetry using thermokinetic parameters. J. Microbiol. Methods 204:106651. doi: 10.1016/j.mimet.2022.106651, PMID: 36503054

[ref26] Bill M. Korsten L. Remize F. Glowacz M. Sivakumar D. (2017). Effect of thyme oil vapours exposure on phenylalanine ammonia-lyase (PAL) and lipoxygenase (LOX) genes expression, and control of anthracnose in ‘Hass’ and ‘Ryan’ avocado fruit. Sci. Hortic. 224, 232–237. doi: 10.1016/j.scienta.2017.06.026

[ref27] Bill M. Sivakumar D. Thompson A. K. Korsten L. (2014). Avocado fruit quality management during the postharvest supply chain. Food Rev. Int. 30, 169–202. doi: 10.1080/87559129.2014.907304

[ref28] Billones-Baaijens R. Úrbez-Torres J. R. Liu M. Ayres M. Sosnowski M. Savocchia S. (2018). Molecular methods to detect and quantify Botryosphaeriaceae Inocula associated with grapevine dieback in Australia. Plant Dis. 102, 1489–1499. doi: 10.1094/PDIS-11-17-1854-RE, PMID: 30673411

[ref29] Bosse R. J. Bower J. P. Bertling I. (2013). Systemic resistance inducers applied preharvest for *Colletotrichum gloeosporioides* control in avocados. Acta Hortic. 1007, 153–160. doi: 10.17660/ActaHortic.2013.1007.14

[ref30] Bowen J. Billing D. Connolly P. Smith W. Cooney J. Burdon J. (2018). Maturity, storage and ripening effects on anti-fungal compounds in the skin of ‘Hass’ avocado fruit. Postharvest Biol. Technol. 146, 43–50. doi: 10.1016/j.postharvbio.2018.08.005

[ref31] Burgess T. I. Tan Y. P. Garnas J. Edwards J. Scarlett K. A. Shuttleworth L. A.. (2019). Current status of the Botryosphaeriaceae in Australia. Australas. Plant Pathol. 48, 35–44. doi: 10.1007/s13313-018-0577-5

[ref32] CABI (2022). Colletotrichum boninense. CABI Int. 1:108203. doi: 10.1079/cabicompendium.108203

[ref33] Cabrera Ardila C. E. Ramirez A. L. Prieto Ortiz F. A. (2020). Spectral analysis for the early detection of anthracnose in fruits of sugar mango (*Mangifera indica*). Comput. Electron. Agric. 173:105357. doi: 10.1016/j.compag.2020.105357

[ref34] Campos-Ferreira U. E. González-Camacho J. M. Carrillo-Salazar A. (2023). Automatic identification of avocado fruit diseases based on machine learning and chromatic descriptors. Rev. Chapingo Ser. Hortic. 29, 115–130. doi: 10.5154/r.rchsh.2023.04.002

[ref35] Cannon P. F. Damm U. Johnston P. R. Weir B. S. (2012). *Colletotrichum*: current status and future directions. Stud. Mycol. 73, 181–213. doi: 10.3114/sim0014, PMID: 23136460 PMC3458418

[ref36] Cao L. Sun X. Dong W. Ma L. Li H. (2023). Detection and quantification of anthracnose pathogen *Colletotrichum fructicola* in cultivated tea-oil *Camellia* species from southern China using a DNA-based qPCR assay. Plant Dis. 107, 363–371. doi: 10.1094/PDIS-04-22-0901-RE, PMID: 35852905

[ref37] Carbone I. Kohn L. M. (1999). A method for designing primer sets for speciation studies in filamentous ascomycetes. Mycologia 91, 553–556. doi: 10.1080/00275514.1999.12061051

[ref38] Casela C. R. Frederiksen R. A. (1993). Survival of *Colletotrichum graminicola* sclerotia in Sorghum stalk residues. Plant Dis. 77, 825–827. doi: 10.1094/PD-77-0825 casel

[ref39] Castro-Moretti F. R. Gentzel I. N. Mackey D. Alonso A. P. (2020). Metabolomics as an emerging tool for the study of plant–pathogen interactions. Meta 10:52. doi: 10.3390/metabo10020052, PMID: 32013104 PMC7074241

[ref40] Cenobio-Galindo A. d. J. Ocampo-López J. Reyes-Munguía A. Carrillo-Inungaray M. L. Cawood M. Medina-Pérez G.. (2019). Influence of bioactive compounds incorporated in a nanoemulsion as coating on avocado fruits (*Persea americana*) during postharvest storage: antioxidant activity, physicochemical changes and structural evaluation. Antioxidants 8:500. doi: 10.3390/antiox8100500 31640249 PMC6826954

[ref41] Chabikwa T. G. Barbier F. F. Tanurdzic M. Beveridge C. A. (2020). *De novo* transcriptome assembly and annotation for gene discovery in avocado, macadamia and mango. Sci Data 7:9. doi: 10.1038/s41597-019-0350-9, PMID: 31913298 PMC6949230

[ref42] Chaplin G. R. Scott K. (1980). Association of calcium in chilling injurychen susceptibility of stored avocados. HortScience 15, 514–515.

[ref43] Chauhan A. De Villiers H. A. C. Meesters L. Paillart M. J. M. Grbović Ž. Panić M.. (2024). Avocado stem-end rot detection using hyperspectral imaging. Acta Hortic. 1396, 107–114. doi: 10.17660/ActaHortic.2024.1396.15

[ref44] Chen L. S. Chu C. Liu C. D. Chen R. S. Tsay J. G. (2006). PCR-based detection and differentiation of anthracnose pathogens, *Colletotrichum gloeosporioides* and *C. truncatum*, from vegetable soybean in Taiwan. J. Phytopathol. 154, 654–662. doi: 10.1111/j.1439-0434.2006.01163.x

[ref45] Chen S. Morgan D. P. Hasey J. K. Anderson K. Michailides T. J. (2014). Phylogeny, morphology, distribution, and pathogenicity of Botryosphaeriaceae and Diaporthaceae from English walnut in California. Plant Dis. 98, 636–652. doi: 10.1094/PDIS-07-13-0706-RE, PMID: 30708543

[ref46] Chikte T. Kopta T. Psota V. Arizmendi J. Chwil M. (2024). A comprehensive review of low- and zero-residue pesticide methods in vegetable production. Agronomy 14:2745. doi: 10.3390/agronomy14112745

[ref47] Chirinos R. Delgado-Pariona J. Aguilar-Galvez A. Figueroa-Merma A. Pacheco-Ávalos A. Campos D.. (2023). Postharvest storage differentially modulates the enzymatic and non-enzymatic antioxidant system of the exocarp and Mesocarp of Hass avocado: implications for disorders. Plants 12:4008. doi: 10.3390/plants12234008, PMID: 38068643 PMC10707783

[ref48] Chung W.-H. Ishii H. Nishimura K. Fukaya M. Yano K. Kajitani Y. (2006). Fungicide sensitivity and phylogenetic relationship of anthracnose Fungi isolated from various fruit crops in Japan. Plant Dis. 90, 506–512. doi: 10.1094/PD-90-0506, PMID: 30786602

[ref49] Clark C. J. McGlone V. A. De Silva H. N. Manning M. A. Burdon J. Mowat A. D. (2004). Prediction of storage disorders of kiwifruit (*Actinidia chinensis*) based on visible-NIR spectral characteristics at harvest. Postharvest Biol. Technol. 32, 147–158. doi: 10.1016/j.postharvbio.2003.11.004

[ref50] Correa-Pacheco Z. N. Bautista-Baños S. Valle-Marquina M. Á. Hernández-López M. (2017). The effect of nanostructured chitosan and chitosan-thyme essential oil coatings on *Colletotrichum gloeosporioides* growth *in vitro* and on cv Hass avocado and fruit quality. J. Phytopathol. 165, 297–305. doi: 10.1111/jph.12562

[ref51] Correa-Pacheco Z. N. Ventura-Aguilar R. I. Zavaleta-Avejar L. Barrera-Necha L. L. Hernández-López M. Bautista-Baños S. (2022). Athracnose disease control and postharvest quality of Hass avocado stored in biobased PLA/PBAT/pine essential oil/chitosan active packaging nets. Plants 11:2278. doi: 10.3390/plants11172278, PMID: 36079660 PMC9460501

[ref52] Cuéllar-Torres E. A. Aguilera-Aguirre S. López-García U. M. Hernández-Oñate M. Á. Montalvo-González E. Ortiz-Basurto R. I.. (2023). Transcriptomic data exploring the effect of Agave Fructans on the induction of the defense system in avocado fruit. PLoS One 18:e0293396. doi: 10.1371/journal.pone.0293396, PMID: 37883423 PMC10602311

[ref53] Cui S. Ma H. Wang X. Yang H. Wu Y. Wei Y.. (2024). Development and comparison of visual LAMP and LAMP-Taq man assays for *Colletotrichum siamense*. Microorganisms 12:1325. doi: 10.3390/microorganisms12071325, PMID: 39065093 PMC11279180

[ref54] Damm U. Baroncelli R. Cai L. Kubo Y. O’Connell R. Weir B.. (2010). *Colletotrichum*: species, ecology and interactions. IMA Fungus 1, 161–165. doi: 10.5598/imafungus.2010.01.02.08, PMID: 22679575 PMC3348780

[ref55] Damm U. Cannon P. F. Liu F. Barreto R. W. Guatimosim E. Crous P. W. (2013). The *Colletotrichum orbiculare* species complex: important pathogens of field crops and weeds. Fungal Divers. 61, 29–59. doi: 10.1007/s13225-013-0255-4

[ref56] Damm U. Cannon P. F. Woudenberg J. H. C. Crous P. W. (2012a). The *Colletotrichum acutatum* species complex. Stud. Mycol. 73, 37–113. doi: 10.3114/sim0010, PMID: 23136458 PMC3458416

[ref57] Damm U. Cannon P. F. Woudenberg J. H. C. Johnston P. R. Weir B. S. Tan Y. P.. (2012b). The *Colletotrichum boninense* species complex. Stud. Mycol. 73, 1–36. doi: 10.3114/sim0002, PMID: 23136457 PMC3458415

[ref58] Damm U. Sato T. Alizadeh A. Groenewald J. Z. Crous P. W. (2019). The *Colletotrichum dracaenophilum, C. magnum* and *C. orchidearum* species complexes. Stud. Mycol. 92, 1–46. doi: 10.1016/j.simyco.2018.04.001, PMID: 29997400 PMC6030544

[ref59] Danderson M. (1986). Omega (Prochloraz), a fungicide for post-harvest control of anthracnose, the *Dothiorella/Colletotrichum* complex and stem-end rot in avocados. S. Afr. Avocado Grow. Assoc. Yearb. 9, 27–30.

[ref60] Dann E. K. Ploetz R. C. Coates L. M. Pegg K. G. (2013). “Foliar, fruit and Soilborne diseases” in The avocado: botany, production and uses. eds. Schaffer B. Wolstenholme B. N. Whiley A. W. (Boston: CABI), 380–422.

[ref61] Defilippi B. Ejsmentewicz T. Covarrubias M. P. Gudenschwager O. Campos-Vargas R. (2018). Changes in cell wall pectins and their relation to postharvest mesocarp softening of “Hass” avocados (*Persea americana* mill.). Plant Physiol. Biochem. 128, 142–151. doi: 10.1016/j.plaphy.2018.05.018, PMID: 29778838

[ref62] Defilippi B. G. Robledo P. Ferreyra R. Soto S. Saavedra J. (2015). Preharvest factors influencing “Hass” avocado (*Persea americana* mill.) quality during long term storage. Acta Hortic. 1071, 137–141. doi: 10.17660/ActaHortic.2015.1071.13

[ref63] Dhakshinamoorthy D. Sundaresan S. Iyadurai A. Subramanian K. S. Janavi G. J. Paliyath G.. (2020). Hexanal vapor induced resistance against major postharvest pathogens of Banana (*Musa acuminata L*.). Plant Pathol. J. 36, 133–147. doi: 10.5423/PPJ.OA.03.2019.0072, PMID: 32296293 PMC7143517

[ref64] Di Stefano V. Avellone G. Bongiorno D. Indelicato S. Massenti R. Lo Bianco R. (2017). Quantitative evaluation of the phenolic profile in fruits of six avocado *(Persea americana)* cultivars by ultra-high-performance liquid chromatography-heated electrospray-mass spectrometry. Int. J. Food Prop. 20, 1302–1312. doi: 10.1080/10942912.2016.1208225

[ref65] Dowling M. Peres N. Villani S. Schnabel G. (2020). Managing *Colletotrichum* on fruit crops: a “complex” challenge. Plant Dis. 104, 2301–2316. doi: 10.1094/PDIS-11-19-2378-FE, PMID: 32689886

[ref66] Elliston J. Kuć J. Williams E. B. Rahe J. E. (1977). Relationship of phytoalexin accumulation to local and systemic protection of bean against anthracnose. J. Phytopathol. 88, 114–130. doi: 10.1111/j.1439-0434.1977.tb03957.x

[ref67] Eskalen A. Faber B. Bianchi M. (2013). Spore trapping and pathogenicity of Fungi in the Botryosphaeriaceae and Diaporthaceae associated with avocado branch canker in California. Plant Dis. 97, 329–332. doi: 10.1094/PDIS-03-12-0260-RE, PMID: 30722352

[ref68] Eskalen A. McDonald V. (2009). Avocado branch canker (formerly Dothiorella canker). California Avocado Soc. 2009 Yearbook 92, 75–84.

[ref69] Estrada A. Dodd J. Jeffries P. (2000). Effect of humidity and temperature on conidial germination and appressorium development of two Philippine isolates of the mango anthracnose pathogen *Colletotrichum gloeosporioides*. Plant Pathol. 49, 608–618. doi: 10.1046/J.1365-3059.2000.00492.X

[ref73] Everett K. Pak H. A. (2001). Orchard survey: effect of preharvest factors on postharvest rots. Avocado Growers Association Annual Research Report 1. Available online at: https://www.avocadosource.com/Journals/NZAGA/NZAGA_2001/NZAGA_2001_03.pdf

[ref74] Everett K. Pak H. A. (2002). Infection criteria for pathogens causing body rots in avocados. Avocado Growers Association Annual Research Report 2. Available online at: https://www.avocadosource.com/Journals/NZAGA/NZAGA_2002/NZAGA_2002_11.pdf

[ref70] Everett K. R. (2020). Avocado diseases affecting fruit quality. CAB Rev. doi: 10.1079/PAVSNNR202015016

[ref71] Everett K. R. Boyd L. M. Pak H. A. Cutting J. G. M. (2007). Calcium, fungicide sprays and canopy density influence postharvest rots of avocado. Australas. Plant Pathol. 36, 22–31. doi: 10.1071/AP06076

[ref77] Everett K. Rees-George J. Johnston P. (2003). Predicting avocado fruit rots by quantifying inoculum potential in the orchard before harvest. V Congreso Mundial del Aguacate. 3, 601–606.

[ref72] Everett K. R. Hallett I. C. Rees-George J. Chynoweth R. W. Pak H. A. (2008). Avocado lenticel damage: the cause and the effect on fruit quality. Postharvest Biol. Technol. 48, 383–390. doi: 10.1016/j.postharvbio.2007.09.008

[ref75] Everett K. R. Pak H. A. Pushparajah I. P. S. Taylor J. T. Astill M. S. King D. B. (2011). Field evaluation of fungicides to control postharvest rots of avocados in New Zealand. N. Z. Plant Prot. 64, 112–118. doi: 10.30843/nzpp.2011.64.5971

[ref76] Everett K. R. Pushparajah I. P. S. Timudo O. E. Ah Chee A. Scheper R. W. A. Shaw P. W.. (2018). Infection criteria, inoculum sources and splash dispersal pattern of *Colletotrichum acutatum* causing bitter rot of apple in New Zealand. Eur. J. Plant Pathol. 152, 367–383. doi: 10.1007/s10658-018-1481-0

[ref78] Evidente A. Punzo B. Andolfi A. Cimmino A. Melck D. Luque J. (2010). Lipophilic phytotoxins produced by *Neofusicoccum parvum*, a grapevine canker agent. Phytopathol. Mediterr. 49, 74–79. doi: 10.14601/Phytopathol_Mediterr-5433

[ref9002] Fang H. Liu X. Dong Y. Feng S. Zhou R. Wang C.. (2021). Transcriptome and proteome analysis of walnut (Juglans regia L.) fruit in response to infection by Colletotrichum gloeosporioides. BMC Plant Biol 21:249. doi: 10.1186/s12870-021-03042-1 34059002 PMC8166054

[ref79] FAO (2024). FAOSTAT. Commodity by country. Available online at: https://www.fao.org/faostat/en/#rankings/countries_by_commodity (Accessed June 14, 2024).

[ref80] Fazari A. Pellicer-Valero O. J. Gómez-Sanchıs J. Bernardi B. Cubero S. Benalia S.. (2021). Application of deep convolutional neural networks for the detection of anthracnose in olives using VIS/NIR hyperspectral images. Comput. Electron. Agric. 187:106252. doi: 10.1016/j.compag.2021.106252

[ref81] Fernandes K. F. D. Queiroga T. S. Lima M. d. C. de Oliveira K. Á. R. Souza E. L. (2024). Interventions based on alternative and sustainable strategies for postharvest control of anthracnose and maintain quality in tropical fruits. Compr. Rev. Food Sci. Food Saf. 23:e13427. doi: 10.1111/1541-4337.13427, PMID: 39137002

[ref82] Fischer I. H. de Moraes M. F. Firmino A. C. Amorim L. (2019). Detection and epidemiological progress of quiescent avocado diseases. Cienc. Rural 49:e20180731. doi: 10.1590/0103-8478cr20180731

[ref83] Fischer I. H. Firmino A. C. (2023). Main diseases of avocado in Brazil. Rev. Anu. Patol. Plantas 29, 106–130. doi: 10.31976/0104-038321v290005

[ref84] Forcelini B. B. Seijo T. E. Amiri A. Peres N. A. (2016). Resistance in strawberry isolates of *Colletotrichum acutatum* from Florida to Quinone-outside inhibitor fungicides. Plant Dis. 100, 2050–2056. doi: 10.1094/PDIS-01-16-0118-RE, PMID: 30683005

[ref85] Fostvedt J. E. Dicks L. Wagstaff C. (2024). Stem end rot infection of avocado (*Persea americana*). Annu. Plant Rev. Online. 7, 1–26. doi: 10.1002/9781119312994.apr0810

[ref86] Freeman S. Katan T. Shabi E. (1998). Characterization of *Colletotrichum* species responsible for anthracnose diseases of various fruits. Plant Dis. 82, 596–605. doi: 10.1094/PDIS.1998.82.6.596, PMID: 30857006

[ref87] Fuentes-Aragón D. Juárez-Vázquez S. B. Vargas-Hernández M. Silva-Rojas H. V. (2018). C*olletotrichum fructicola*, a member of *Colletotrichum gloeosporioides sensu lato*, is the causal agent of anthracnose and soft rot in avocado fruits cv. Hass. Mycobiology 46, 92–100. doi: 10.1080/12298093.2018.1454010, PMID: 29963310 PMC6023250

[ref88] Fuentes-Aragón D. Silva-Rojas H. V. Guarnaccia V. Mora-Aguilera J. A. Aranda-Ocampo S. Bautista-Martínez N.. (2020). *Colletotrichum* species causing anthracnose on avocado fruit in Mexico: current status. Plant Pathol. 69, 1513–1528. doi: 10.1111/ppa.13234

[ref89] Fussell R. J. (2016). An overview of regulation and control of pesticide residues in food. Thermo fisher scientific White. Available online at: https://www.thermofisher.com/blog/food/an-overview-of-regulation-and-control-of-pesticide-residues-in-food/

[ref90] Galsurker O. Diskin S. Maurer D. Feygenberg O. Alkan N. (2018). Fruit stem-end rot. Horticulturae 4:50. doi: 10.3390/horticulturae4040050 PMC7232454 32295088

[ref91] Gamble J. Harker F. R. Jaeger S. R. White A. Bava C. Beresford M.. (2010). The impact of dry matter, ripeness and internal defects on consumer perceptions of avocado quality and intentions to purchase. Postharvest Biol. Technol. 57, 35–43. doi: 10.1016/j.postharvbio.2010.01.001

[ref92] Gan P. Hiroyama R. Tsushima A. Masuda S. Shibata A. Ueno A.. (2021). Telomeres and a repeat-rich chromosome encode effector gene clusters in plant pathogenic *Colletotrichum* fungi. Environ. Microbiol. 23, 6004–6018. doi: 10.1111/1462-2920.15490, PMID: 33780109

[ref93] Gan P. Ikeda K. Irieda H. Narusaka M. O'Connell R. J. Narusaka Y.. (2013). Comparative genomic and transcriptomic analyses reveal the hemibiotrophic stage shift of *Colletotrichum* fungi. New Phytol. 197, 1236–1249. doi: 10.1111/nph.12085, PMID: 23252678

[ref94] Garg N. Ahmad F. J. Kar S. (2022). Recent advances in loop-mediated isothermal amplification (lamp) for rapid and efficient detection of pathogens. Curr. Res. Microb. Sci. 3:100120. doi: 10.1016/j.crmicr.2022.100120, PMID: 35909594 PMC9325740

[ref95] Garrido C. Carbú M. Fernández-Acero F. J. Boonham N. Colyer A. Cantoral J. M.. (2009). Development of protocols for detection of *Colletotrichum acutatum* and monitoring of strawberry anthracnose using real-time PCR. Plant Pathol. 58, 43–51. doi: 10.1111/j.1365-3059.2008.01933.x

[ref96] Giblin F. R. Tan Y. P. Mitchell R. Coates L. M. Irwin J. A. G. Shivas R. G. (2018). *Colletotrichum* species associated with pre- and post-harvest diseases of avocado and mango in eastern Australia. Australas. Plant Pathol. 47, 269–276. doi: 10.1007/s13313-018-0553-0

[ref97] Giuggioli N. R. Merlino V. M. Sparacino A. Peano C. Borra D. Massaglia S. (2023). Customer preferences heterogeneity toward avocado: a latent class approach based on the best–worst scaling choice modeling. Agric. Food Econ. 11:46. doi: 10.1186/s40100-023-00289-0

[ref98] Glass N. L. Donaldson G. C. (1995). Development of primer sets designed for use with the PCR to amplify conserved genes from filamentous ascomycetes. Appl. Environ. Microbiol. 61, 1323–1330. doi: 10.1128/aem.61.4.1323-1330.1995, PMID: 7747954 PMC167388

[ref99] Gong D. Bi Y. Li Y. Zong Y. Han Y. Prusky D. (2019). Both *Penicillium expansum* and *Trichothecim roseum* infections promote the ripening of apples and release specific volatile compounds. Front. Plant Sci. 10:338. doi: 10.3389/fpls.2019.00338, PMID: 30949192 PMC6435981

[ref100] Gong D. Bi Y. Zong Y. Li Y. Sionov E. Prusky D. (2022). Characterization and sources of volatile organic compounds produced by postharvest pathogenic fungi colonized fruit. Postharvest Biol. Technol. 188:111903. doi: 10.1016/j.postharvbio.2022.111903

[ref101] González-Gutiérrez K. N. Ragazzo-Sánchez J. A. Calderón-Santoyo M. (2024a). Field and postharvest application of microencapsulated *Yamadazyma mexicana* Lpa14: anthracnose control and effect on postharvest quality in avocado (*Persea americana* mill. Cv. Hass). Pest Manag. Sci. 80, 3459–3469. doi: 10.1002/ps.8052 38415946

[ref102] González-Gutiérrez K. N. Ragazzo-Sánchez J. A. Calderón-Santoyo M. (2024b). Bioformulation of *Yamadazyma mexicana* LPa14 by electrospraying process: anthracnose control and effect on postharvest quality of avocado fruit. Biol. Control 190:105449. doi: 10.1016/j.biocontrol.2024.105449 38415946

[ref103] Guarnaccia V. Vitale A. Cirvilleri G. Aiello D. Susca A. Epifani F.. (2016). Characterisation and pathogenicity of fungal species associated with branch cankers and stem-end rot of avocado in Italy. Eur. J. Plant Pathol. 146, 963–976. doi: 10.1007/s10658-016-0973-z

[ref104] Guetsky R. Kobiler I. Wang X. Perlman N. Gollop N. Avila-Quezada G.. (2005). Metabolism of the flavonoid epicatechin by laccase of *Colletotrichum gloeosporioides* and its effect on pathogenicity on avocado fruits. Phytopathology 95, 1341–1348. doi: 10.1094/PHYTO-95-1341, PMID: 18943366

[ref105] Gurr S. (2021). Integrated disease management strategies. Eduzone Int. Peer Rev. Refer. Multidis. J. 10, 1114–1119.

[ref106] Harker F. R. White A. Beresford M. Wohlers M. Corbett T. Hofman P.. (2010). Shepard avocado maturity consumer sensory research. Horticulture Australia. Available online at: https://www.avocado.org.au/wp-content/uploads/2016/12/AV09026-Shepard-Avocado-Maturity-Consumer-Sensory-Research.pdf

[ref107] Hartill W. F. T. (1991). Post-harvest diseases of avocado fruits in New Zealand. N. Z. J. Crop. Hortic. Sci. 19, 297–304. doi: 10.1080/01140671.1991.10421814

[ref108] Hartill W. F. T. Everett K. R. (2002). Inoculum sources and infection pathways of pathogens causing stem-end rots of ‘Hass’ avocado (*Persea americana*). N. Z. J. Crop. Hortic. Sci. 30, 249–260. doi: 10.1080/01140671.2002.9514221

[ref109] Hass R. (1935). Hass Avocado Patent. United States Patent and Trademark Office. Available online at: https://www.avocadosource.com/links/hasspatent_1935.pdf

[ref110] Hattori Y. Nakashima C. Kitabata S. Naito K. Hieno A. Alvarez L. V.. (2021). Identification of the *Colletotrichum* species associated with mango diseases and a universal lamp detection method for *C. gloeosporioides* species complex. Plant Fungal Res. 4, 2–13. doi: 10.30546/2664-5297.2021.1.49

[ref9003] Hayes C. N. Nakahara H. Ono A. Tsuge M. Oka S. (2024). From Omics to Multi-Omics: A Review of Advantages and Tradeoffs. Genes 15:1551. doi: 10.3390/genes15121551 39766818 PMC11675490

[ref111] He D. Zhan J. Xie L. (2016). Problems, challenges and future of plant disease management: from an ecological point of view. J. Integr. Agric. 15, 705–715. doi: 10.1016/S2095-3119(15)61300-4

[ref112] Hernández-Lauzardo A. N. Campos-Martínez A. Velázquez-del Valle M. G. Flores-Moctezuma H. E. Suárez-Rodríguez R. Ramírez-Trujillo J. A. (2015). First report of *Colletotrichum godetiae* causing anthracnose on avocado in Mexico. Plant Dis. 99:555. doi: 10.1094/PDIS-10-14-1019-PDN

[ref113] Herrera-González J. (2017). Aplicaciones de Fungicidas en Precosecha que Controlan Enfermedades Postcosecha de Aguacate ‘Hass’ En Michoacán. Memorias del V Congreso Latinoamericano del Aguacate. Available online at: http://www.avocadosource.com/journals/memorias_vcla/2017/memorias_vcla_2017_pg_126.pdf

[ref114] Herrera-González J. A. Bautista-Baños S. Serrano M. Romanazzi G. Gutiérrez-Martínez P. (2021). Non-chemical treatments for the pre- and post-harvest elicitation of defense mechanisms in the fungi–avocado pathosystem. Molecules 26:6819. doi: 10.3390/molecules26226819, PMID: 34833910 PMC8617955

[ref115] Hershkovitz V. Friedman H. Goldschmidt E. E. Pesis E. (2009). The role of the embryo and ethylene in avocado fruit Mesocarp discoloration. J. Exp. Bot. 60, 791–799. doi: 10.1093/jxb/ern328, PMID: 19196750 PMC2652053

[ref116] Ibarra-Laclette E. Méndez-Bravo A. Pérez-Torres C. A. Albert V. A. Mockaitis K. Kilaru A.. (2015). Deep sequencing of the Mexican avocado transcriptome, an ancient angiosperm with a high content of fatty acids. BMC Genomics 16:599. doi: 10.1186/s12864-015-1775-y, PMID: 26268848 PMC4533766

[ref117] Iñiguez-Moreno M. González-Gutiérrez K. N. Ragazzo-Sánchez J. A. Narváez-Zapata J. A. Sandoval-Contreras T. Calderón-Santoyo M. (2021). Morphological and molecular identification of the causal agents of post-harvest diseases in avocado fruit, and potential biocontrol with *Meyerozyma caribbica*. Arch. Phytopathol. Plant Protect. 54, 411–430. doi: 10.1080/03235408.2020.1834806

[ref118] Jansen R. M. C. Wildt J. Kappers I. F. Bouwmeester H. J. Hofstee J. W. van Henten E. J. (2011). Detection of diseased plants by analysis of volatile organic compound emission. Annu. Rev. Phytopathol. 49, 157–174. doi: 10.1146/annurev-phyto-072910-095227, PMID: 21663436

[ref119] Jeong H. Yoon J. Park H. Son M. Park S.-Y. Kim K.-H. (2024). Spore PCR and qPCR methods for rapid detection of five *Colletotrichum* species responsible for pepper anthracnose in Korea. Res. Plant Dis. 30, 219–228. doi: 10.5423/RPD.2024.30.3.219

[ref120] Jiang L. Wu P. Yang L. Liu C. Guo P. Wang H.. (2021). Transcriptomics and metabolomics reveal the induction of flavonoid biosynthesis pathway in the interaction of *Stylosanthes-Colletotrichum gloeosporioides*. Genomics 113, 2702–2716. doi: 10.1016/j.ygeno.2021.06.004, PMID: 34111523

[ref121] Jiménez-Ariza N. V. Soto-Heredia J. M. Casas-Díaz A. V. Aragón-Caballero L. M. (2023). Posible Inducción de Resistencia Sistémica a *Lasiodiplodia theobromae* en Aguacate (*Persea americana* Mill.) en Condiciones Semicontroladas en La Molina. Peruv. J. Agron. 7, 132–143. doi: 10.21704/pja.v7i2.2053

[ref122] Johnson G. I. Mead A. J. Cooke A. W. Dean J. R. (1992). Mango stem end rot pathogens - fruit infection by endophytic colonisation of the inflorescence and pedicel. Ann. Appl. Biol. 120, 225–234. doi: 10.1111/j.1744-7348.1992.tb03420.x

[ref123] Kamali Dashtarzhaneh M. Liu L. Pegahrad Z. Valencia Bernal V. Du K. Khodadadi F. (2025). Detection and quantification of avocado sunblotch viroid in California avocado orchards using digital loop-mediated amplification and droplet digital PCR. Phytopathology 115, 1051–1064. doi: 10.1094/PHYTO-02-25-0064-R, PMID: 40280875

[ref124] Karim M. M. Usman H. M. Tan Q. Hu J.-J. Fan F. Hussain R.. (2024). Fungicide resistance in *Colletotrichum fructicola* and *Colletotrichum siamense* causing peach anthracnose in China. Pestic. Biochem. Physiol. 203:106006. doi: 10.1016/j.pestbp.2024.106006 39084801

[ref125] Karunanayake K. O. L. C. Adikaram N. K. B. (2020). Stem-end rot in major tropical and sub-tropical fruit species. Ceylon J. Sci. 49, 327–336. doi: 10.4038/cjs.v49i5.7800

[ref126] Karunanayake K. O. L. C. Liyanage K. C. M. Jayakody L. K. R. R. Somaratne S. (2020). Basil oil incorporated beeswax coating to increase shelf life and reduce anthracnose development in mango cv. Willard. Ceylon J. Sci. 49, 355–361. doi: 10.4038/cjs.v49i5.7802

[ref127] Kassim A. Seyoum Workneh T. Bezuidenhout C. (2013). A review on postharvest handling of avocado fruit. Afr. J. Agric. Res. 8, 2385–2402.

[ref128] Kende H. (1993). Ethylene biosynthesis. Annu. Rev. Plant Biol. 44, 283–307. doi: 10.1146/annurev.pp.44.060193.001435

[ref129] Khlaif S. Mudalal S. Ruiz-Canales A. Abu-Khalaf N. (2024). Electronic nose for detecting *Colletotrichum coccodes* causing anthracnose fruit rots in tomatoes. Smart Agric. Technol. 8:100451. doi: 10.1016/j.atech.2024.100451

[ref130] Khodadadi F. González J. B. Martin P. L. Giroux E. Bilodeau G. J. Peter K. A.. (2020). Identification and characterization of Colletotrichum species causing apple bitter rot in New York and description of C. noveboracense sp. nov. Sci. Rep. 10:11043. doi: 10.1038/s41598-020-66761-9, PMID: 32632221 PMC7338416

[ref131] Khodadadi F. Santander R. D. McHenry D. J. Jurick W. M. Aćimović S. G. (2023). A bitter, complex problem: causal *Colletotrichum* species in Virginia orchards and apple fruit susceptibility. Plant Dis. 107, 3164–3175. doi: 10.1094/PDIS-12-22-2947-RE, PMID: 37102728

[ref132] Kimaru S. K. Monda E. Cheruiyot R. C. Mbaka J. Alakonya A. (2018). Sensitivity of *Colletotrichum gloeosporioides* isolates from diseased avocado fruits to selected fungicides in Kenya. Adv. Agric. 2018:3567161. doi: 10.1155/2018/3567161

[ref133] King K. M. Canning G. G. M. West J. S. (2024). MinION sequencing of fungi in sub-Saharan African air and a novel LAMP assay for rapid detection of the tropical Phytopathogenic genus *Lasiodiplodia*. Pathogens 13:330. doi: 10.3390/pathogens13040330, PMID: 38668285 PMC11053906

[ref134] Kirkby E. A. Pilbeam D. J. (1984). Calcium as a plant nutrient. Plant Cell Environ. 7, 397–405. doi: 10.1111/j.1365-3040.1984.tb01429.x

[ref135] Koo-Lee S. (2024). Harvesting avocados. University of California Cooperative Extension Ventura. Available online at: https://ceventura.ucanr.edu/Com_Ag/Subtropical/Avocado_Handbook/Harvesting/Harvesting_Avocados_/

[ref136] Korsten G. (1997). Market Survey of Stem-end Rot and Anthracnose on Fuerte Avocados and Comparison of Colletotrichum gloeosporioides Isolates from Different Avocado Producing areas. SAAG. 20, 101–105.

[ref137] Kwon J.-H. Choi O. Lee Y. Kim S. Kang B. Kim J. (2020). Anthracnose on postharvest avocado caused by *Colletotrichum kahawae subsp. ciggaro* in South Korea. Can. J. Plant Pathol. 42, 508–513. doi: 10.1080/07060661.2019.1696891

[ref138] Lan C. Yao J. Yang X. Ruan H. Yu D. Jiang J. (2020). Specific and sensitive detection of the guava fruit anthracnose pathogen (*Colletotrichum gloeosporioides*) by loop-mediated isothermal amplification (LAMP) assay. Can. J. Microbiol. 66, 17–24. doi: 10.1139/cjm-2019-0099, PMID: 31553892

[ref139] Law J. W.-F. Ab Mutalib N.-S. Chan K.-G. Lee L.-H. (2015). Rapid methods for the detection of foodborne bacterial pathogens: principles, applications, advantages and limitations. Front. Microbiol. 5:770. doi: 10.3389/fmicb.2014.00770, PMID: 25628612 PMC4290631

[ref140] Lelièvre J.-M. Latchè A. Jones B. Bouzayen M. Pech J.-C. (1997). Ethylene and fruit ripening. Physiol. Plant. 101, 727–739. doi: 10.1111/j.1399-3054.1997.tb01057.x

[ref141] Leskovac A. Petrović S. (2023). Pesticide use and degradation strategies: food safety, challenges and perspectives. Foods 12:2709. doi: 10.3390/foods12142709, PMID: 37509801 PMC10379487

[ref142] Li L. Mohd M. H. Mohamed Nor N. M. I. Subramaniam S. Latiffah Z. (2021). Identification of Botryosphaeriaceae associated with stem-end rot of mango (*Mangifera indica* L.) in Malaysia. J. Appl. Microbiol. 130, 1273–1284. doi: 10.1111/jam.14828, PMID: 32813902

[ref143] Liang D. Jiang Y. Zhang Y. Mao C. Ma T. Zhang C. (2024). The comparative genomics of Botryosphaeriaceae suggests gene families of *Botryosphaeria dothidea* related to pathogenicity on Chinese hickory tree. J. Fungi 10:299. doi: 10.3390/jof10040299 PMC11051394 38667970

[ref9006] Liang X. Shang S. Dong Q. Wang B. Zhang R. Gleason M. L.. (2018). Transcriptomic analysis reveals candidate genes regulating development and host interactions of *Colletotrichum fructicola*. BMC Genomics 19:557. doi: 10.1186/s12864-018-4934-0 30055574 PMC6064131

[ref144] Liu J.-K. Phookamsak R. Doilom M. Wikee S. Li Y.-M. Ariyawansha H.. (2012). Towards a natural classification of Botryosphaeriales. Fungal Divers. 57, 149–210. doi: 10.1007/s13225-012-0207-4

[ref145] Llanos A. Apaza W. (2021). Distribution of stem-end rot on the canopy in ‘Hass’ avocado trees in two coastal areas in Peru. Peruv. J. Agron. 5, 60–70. doi: 10.21704/pja.v5i2.1771

[ref146] López-Cobo A. Gómez-Caravaca A. M. Pasini F. Caboni M. F. Segura-Carretero A. Fernández-Gutiérrez A. (2016). HPLC-DAD-ESI-QTOF-MS and HPLC-FLD-MS as valuable tools for the determination of phenolic and other polar compounds in the edible part and by-products of avocado. LWT 73, 505–513. doi: 10.1016/j.lwt.2016.06.049

[ref147] Lyu X. Agar O. T. Barrow C. J. Dunshea F. R. Suleria H. A. R. (2023). Phenolic compounds profiling and their antioxidant capacity in the peel, pulp, and seed of Australian grown avocado. Antioxidants 12:185. doi: 10.3390/antiox12010185, PMID: 36671046 PMC9855119

[ref148] Madhu G. S. Rani A. T. Muralidhara B. M. Deepak G. N. Rajendiran S. Manjunatha L.. (2025). Diversity, phylogeny, and pathogenicity of *Lasiodiplodia* spp. infecting avocado in India and development of sensitive point-of-care LAMP assay for detection of *Lasiodiplodia pseudotheobromae*. Physiol. Mol. Plant Pathol. 138:102687. doi: 10.1016/j.pmpp.2025.102687

[ref149] Magallón-Andalón C. G. Calderón-Santoyo M. Balois-Morales R. Ochoa-Jiménez V. A. Casas-Junco P. P. López-Guzmán G. G.. (2025). Exploring the biocontrol potential of *Bacillus thuringiensis* against *Colletotrichum gloeosporioides* in “Hass” avocado fruits. Physiol. Mol. Plant Pathol. 138:102724. doi: 10.1016/j.pmpp.2025.102724

[ref150] Matsui T. Sugimori H. Koseki S. Koyama K. (2023). Automated detection of internal fruit rot in Hass avocado via deep learning-based semantic segmentation of X-ray images. Postharvest Biol. Technol. 203:112390. doi: 10.1016/j.postharvbio.2023.112390

[ref151] Mazhar M. Joyce D. Hofman P. Vu N. (2018). Factors contributing to increased bruise expression in avocado (*Persea americana* M.) cv. ‘Hass’ fruit. Postharvest Biol. Technol. 143, 58–67. doi: 10.1016/j.postharvbio.2018.04.015

[ref9004] McDonald V. Lynch S. Eskalen A. (2009). First report of *Neofusicoccum australe*, *N. luteum*, and *N. parvum* associated with avocado branch canker in California. Plant Dis. 93:967. doi: 10.1094/PDIS-93-9-0967B 30754556

[ref152] McHenry D. J. Aćimović S. G. (2024). New species-specific real-time PCR assays for *Colletotrichum* species causing bitter rot of apple. Microorganisms 12:878. doi: 10.3390/microorganisms12050878, PMID: 38792708 PMC11123832

[ref153] Messenger B. Menge J. Amrhein C. Faber B. (1997). The effects of calcium on avocado growth and root health. Calif. Avocado Soc. 1997 Yearb. 81, 69–78.

[ref154] Moalemiyan M. Vikram A. Kushalappa A. C. Yaylayan V. (2006). Volatile metabolite profiling to detect and discriminate stem-end rot and anthracnose diseases of mango fruits. Plant Pathol. 55, 792–802. doi: 10.1111/j.1365-3059.2006.01443.x

[ref155] Möller H. Slippers B. van den Berg N. (2025). Branch canker battles: understanding and managing the Botryosphaeriaceae in avocado. Phytoparasitica 53:17. doi: 10.1007/s12600-024-01227-6

[ref156] Moral J. Morgan D. Trapero A. Michailides T. J. (2019). Ecology and epidemiology of diseases of nut crops and olives caused by Botryosphaeriaceae Fungi in California and Spain. Plant Dis. 103, 1809–1827. doi: 10.1094/PDIS-03-19-0622-FE, PMID: 31232653

[ref157] Moriwaki J. Sato T. Tsukiboshi T. (2003). Morphological and molecular characterization of *Colletotrichum boninense* sp. nov. from Japan. Mycoscience 44, 0047–0053. doi: 10.1007/s10267-002-0079-7

[ref158] Morse J. G. Faber B. A. (2017). UC IPM pest management guidelines: avocado. University of California Agriculture and Natural Resources, Statewide IPM Program. Available online at: http://ipm.ucanr.edu/PMG/selectnewpest.avocado.html

[ref159] Mpho M. Sivakumar D. Sellamuthu P. S. Bautista-Baños S. (2013). Use of lemongrass oil and modified atmosphere packaging on control of anthracnose and quality maintenance in avocado cultivars. J. Food Qual. 36, 198–208. doi: 10.1111/jfq.12027

[ref160] Munhuweyi K. Mpai S. Sivakumar D. (2020). Extension of avocado fruit postharvest quality using non-chemical treatments. Agronomy 10:212. doi: 10.3390/agronomy10020212

[ref161] Nagel J. H. Wingfield M. J. Slippers B. (2021a). Increased abundance of secreted hydrolytic enzymes and secondary metabolite gene clusters define the genomes of latent plant pathogens in the Botryosphaeriaceae. BMC Genomics 22:589. doi: 10.1186/s12864-021-07902-w, PMID: 34348651 PMC8336260

[ref162] Nagel J. H. Wingfield M. J. Slippers B. (2021b). Next-generation sequencing provides important insights into the biology and evolution of the Botryosphaeriaceae. Fungal Biol. Rev. 38, 25–43. doi: 10.1016/j.fbr.2021.09.002

[ref163] Navarro B. L. Edwards Molina J. P. Nogueira Júnior A. F. (2022). Penetration by Botryosphaeriaceae species in avocado, guava and persimmon fruit during postharvest. J. Phytopathol. 170, 57–68. doi: 10.1111/jph.13055

[ref164] Ni H.-F. Yang H.-R. Chen R.-S. Hung T.-H. Liou R.-F. (2012). A nested multiplex PCR for species-specific identification and detection of Botryosphaeriaceae species on mango. Eur. J. Plant Pathol. 133, 819–828. doi: 10.1007/s10658-012-0003-8

[ref165] Nilmini R. K. Kodituwakku T. D. Abeywickrama K. Kuruppu M. (2021). In vitro and in vivo application of eco-friendly treatments to control postharvest stem-end rot of naturally infected avocado (cv. Pollock). J. Agric. Sci. (Sri Lanka) 16, 283–299. doi: 10.4038/jas.v16i2.9335

[ref166] Núñez-Lillo G. Ponce E. Arancibia-Guerra C. Carpentier S. Carrasco-Pancorbo A. Olmo-García L.. (2023). A multiomics integrative analysis of color de-synchronization with softening of ‘Hass’ avocado fruit: a first insight into a complex physiological disorder. Food Chem. 408:135215. doi: 10.1016/j.foodchem.2022.135215, PMID: 36528992

[ref167] O’Brien C. Alamar M. C. (2025). An overview of non-destructive technologies for postharvest quality assessment in horticultural crops. J. Hortic. Sci. Biotechnol. 1–9. doi: 10.1080/14620316.2025.2489974

[ref9007] O’Connell R. J. Thon M. R. Hacquard S. Amyotte S. G. Kleemann J. Torres M. F.. (2012). Lifestyle transitions in plant pathogenic Colletotrichum fungi deciphered by genome and transcriptome analyses. Nat Genet 44:1060–1065. doi: 10.1038/ng.2372 22885923 PMC9754331

[ref168] O’Donnell K. Cigelnik E. (1997). Two divergent intragenomic rDNA ITS2 types within a monophyletic lineage of the fungus *Fusarium* are nonorthologous. Mol. Phylogenet. Evol. 7, 103–116. doi: 10.1006/mpev.1996.0376 9007025

[ref169] Obenland D. Collin S. Sievert J. Negm F. Arpaia M. L. (2012). Influence of maturity and ripening on aroma volatiles and flavor in ‘Hass’ avocado. Postharvest Biol. Technol. 71, 41–50. doi: 10.1016/j.postharvbio.2012.03.006

[ref170] Obianom C. Romanazzi G. Sivakumar D. (2019). Effects of chitosan treatment on avocado postharvest diseases and expression of phenylalanine ammonia-lyase, chitinase and lipoxygenase genes. Postharvest Biol. Technol. 147, 214–221. doi: 10.1016/j.postharvbio.2018.10.004

[ref171] Ochoa S. Vázquez G. (2006). Stem-end-rot of avocado (*Persea americana* mill. cv Hass) incidence at Michoacán, México. In VI world avocado congress. Available online at: http://www.avocadosource.com/wac6/en/Resumen/4a-178.pdf

[ref172] Olivares D. García-Rojas M. Ulloa P. A. Riveros A. Pedreschi R. Campos-Vargas R.. (2022). Response mechanisms of “Hass” avocado to sequential 1–methylcyclopropene applications at different maturity stages during cold storage. Plants 11:1781. doi: 10.3390/plants11131781, PMID: 35807733 PMC9269533

[ref9008] Ozbudak E. Carrillo-Tarazona Y. Diaz E. A. Zambon F. T. Rossi L. Peres N. A.. (2025). Transcriptome analysis of Colletotrichum nymphaeae-Strawberry interaction reveals in planta expressed genes associated with virulence. Front. Plant Sci. 15:1390926. doi: 10.3389/fpls.2024.1390926 39925370 PMC11803528

[ref173] Pak H. Dixon J. Smith D. Elmsly T. Cutting J. (2003). Impact of rainfall prior to harvest on ripe fruit quality of ‘Hass’ avocados in New Zealand. In Proceedings V world avocado congress (Actas V Congreso Mundial del Aguacate), 629–634

[ref174] Pale M. Pérez-Torres C.-A. Arenas-Huertero C. Villafán E. Sánchez-Rangel D. Ibarra-Laclette E. (2024). Genome-wide transcriptional response of avocado to *Fusarium* sp. infection. Plants 13:2886. doi: 10.3390/plants13202886, PMID: 39458832 PMC11511450

[ref175] Pandey A. Sain S. K. Singh P. (2016). A perspective on integrated disease management in agriculture. Bio Bulletin 2, 13–29.

[ref9009] Paolinelli-Alfonso M. Villalobos-Escobedo J. M. Rolshausen P. Herrera-Estrella A. Galindo-Sánchez C. López-Hernández J. F.. (2016). Global transcriptional analysis suggests Lasiodiplodia theobromae pathogenicity factors involved in modulation of grapevine defensive response. BMC Genom. 17:615. doi: 10.1186/s12864-016-2952-3 PMC4981995 27514986

[ref176] Parthasarathy S. Thiribhuvanamala G. Subramanian K. S. Paliyath G. Jayasankar S. Prabakar K. (2017). Volatile metabolites fingerprinting to discriminate the major post harvest diseases of mango caused by *Colletotrichum gloeosporioides* Penz. and L*asiodiplodia theobromae* pat. Ann. Phytomed. Int. J. 6, 55–62. doi: 10.21276/ap.2017.6.2.4

[ref177] Partridge C. J. Pak H. A. Brookbanks P. (2002). An investigation into the effects of pre- harvest sprays of calcium-containing formulations in reducing post-harvest rots in “Hass” avocados. NZ Avocado Growers Assoc. Ann. Res. Rep. 2, 1–6.

[ref178] Patel M. K. Maurer D. Feyngenberg O. Duanis-Assaf D. Sela N. Ovadia R.. (2023). Revealing the mode of action of phenylalanine application in inducing fruit resistance to fungal pathogens. Postharvest Biol. Technol. 199:112298. doi: 10.1016/j.postharvbio.2023.112298

[ref9010] Peng J. Wang X. Abeywickrama P. D. Guo H. Liu M. Xing Q.. (2025). Transcriptomic analyses of LtEpg1-and VvKINβ1-transgenic plants in response to Lasiodiplodia theobromae infection. Physiol. Mol. Plant Pathol. 139:102812. doi: 10.1016/j.pmpp.2025.102812

[ref179] Penter M. Stassen P. (2000). The effect of pre- and postharvest calcium applications on the postharvest quality of Pinkerton avocados. S. Afr. Avocado Grow. Assoc. Yearb. 23, 1–7.

[ref180] Peralta-Ruiz Y. Rossi C. Grande-Tovar C. D. Chaves-López C. (2023). Green management of postharvest anthracnose caused by *Colletotrichum gloeosporioides*. J. Fungi 9:623. doi: 10.3390/jof9060623, PMID: 37367558 PMC10302910

[ref181] Perfect S. E. Hughes H. B. O’Connell R. J. Green J. R. (1999). *Colletotrichum:* a model genus for studies on pathology and fungal–plant interactions. Fungal Genet. Biol. 27, 186–198. doi: 10.1006/fgbi.1999.1143 10441444

[ref182] Phillips A. J. L. Alves A. Abdollahzadeh J. Slippers B. Wingfield M. J. Groenewald J. Z.. (2013). The Botryosphaeriaceae: genera and species known from culture. Stud. Mycol. 76, 51–167. doi: 10.3114/sim0021, PMID: 24302790 PMC3825232

[ref183] Phillips A. J. L. Hyde K. D. Alves A. Liu J.-K. (2019). Families in Botryosphaeriales: a phylogenetic, morphological and evolutionary perspective. Fungal Divers. 94, 1–22. doi: 10.1007/s13225-018-0416-6

[ref184] Prusky D. Alkan N. Mengiste T. Fluhr R. (2013). Quiescent and necrotrophic lifestyle choice during postharvest disease development. Annu. Rev. Phytopathol. 51, 155–176. doi: 10.1146/annurev-phyto-082712-102349, PMID: 23682917

[ref185] Prusky D. Gold S. Keen N. T. (1989). Purification and characterization of an endopolygalacturonase produced by *Colletotrichum gloeosporioides*. Physiol. Mol. Plant Pathol. 35, 121–133. doi: 10.1016/0885-5765(89)90082-9

[ref186] Prusky D. Lichter A. (2007). Activation of quiescent infections by postharvest pathogens during transition from the biotrophic to the necrotrophic stage. Feder. Eur. Microbiol. Soc. 268, 1–8. doi: 10.1111/j.1574-6968.2006.00603.x, PMID: 17227463

[ref187] Qiu F. Xu G. Zhou J. Zheng F. Q. Zheng L. Miao W. G.. (2020). First report of *Botryosphaeria dothidea* causing stem-end rot in avocado (*Persea americana*) in China. Plant Dis. 104:286. doi: 10.1094/PDIS-07-19-1439-PDN

[ref188] Rahman M. Islam T. Schwegel R. Louws F. J. (2019). Simultaneous detection of *Colletotrichum acutatum* and *C. Gloeosporioides* from quiescently infected strawberry foliage by real-time PCR based on high resolution melt curve analysis. Am. J. Plant Sci. 10, 382–401. doi: 10.4236/ajps.2019.103028

[ref189] Ramírez-Gil J. G. Henao-Rojas J. C. Morales-Osorio J. G. (2021). Postharvest diseases and disorders in avocado cv. Hass and their relationship to Preharvest management practices. Heliyon 7:e05905. doi: 10.1016/j.heliyon.2021.e05905, PMID: 33490674 PMC7809187

[ref190] Ramírez-Gil J. G. López J. H. Henao-Rojas J. C. (2020). Causes of Hass avocado fruit rejection in preharvest, harvest, and packinghouse: economic losses and associated variables. Agronomy 10:8. doi: 10.3390/agronomy10010008

[ref191] Ramos-Aguilar A. L. Ornelas-Paz J. Tapia-Vargas L. M. Gardea-Bejar A. A. Yahia E. M. Ornelas-Paz J. J.. (2021). Metabolomic analysis and physical attributes of ripe fruits from Mexican creole (*Persea americana var. drymifolia*) and “Hass” avocados. Food Chem. 354:129571. doi: 10.1016/j.foodchem.2021.129571, PMID: 33761337

[ref192] Regnier T. Combrinck S. du Plooy W. Botha B. (2010). Evaluation of *Lippia scaberrima* essential oil and some pure terpenoid constituents as postharvest mycobiocides for avocado fruit. Postharvest Biol. Technol. 57, 176–182. doi: 10.1016/j.postharvbio.2010.03.010

[ref193] Rivera S. A. Ferreyra R. Robledo P. Selles G. Arpaia M. L. Saavedra J.. (2017). Identification of Preharvest factors determining postharvest ripening behaviors in ‘Hass’ avocado under long term storage. Sci. Hortic. 216, 29–37. doi: 10.1016/j.scienta.2016.12.024

[ref194] Rojas E. I. Rehner S. A. Samuels G. J. Van Bael S. A. Herre E. A. Cannon P.. (2010). *Colletotrichum gloeosporioides* s.l. associated with *Theobroma cacao* and other plants in Panamá: multilocus phylogenies distinguish host-associated pathogens from asymptomatic endophytes. Mycologia 102, 1318–1338. doi: 10.3852/09-244, PMID: 20943565

[ref195] Rolando J. C. Jue E. Barlow J. T. Ismagilov R. F. (2020). Real-time kinetics and high-resolution melt curves in single-molecule digital lamp to differentiate and study specific and non-specific amplification. Nucleic Acids Res. 48:e42. doi: 10.1093/nar/gkaa099, PMID: 32103255 PMC7144905

[ref196] Romero-Cuadrado L. Aguado A. Ruano-Rosa D. Capote N. (2024). Triplex real-time qPCR for the simultaneous detection of Botryosphaeriaceae species in Woody crops and environmental samples. Front. Plant Sci. 15:1435462. doi: 10.3389/fpls.2024.1435462, PMID: 39464288 PMC11502354

[ref197] Romero-Cuadrado L. López-Herrera C. J. Aguado A. Capote N. (2023). Duplex real-time PCR assays for the simultaneous detection and quantification of Botryosphaeriaceae species causing canker diseases in Woody crops. Plants 12:2205. doi: 10.3390/plants12112205, PMID: 37299184 PMC10255876

[ref198] Rosado A. W. C. Machado A. R. Freire F. d. C. O. Pereira O. L. (2016). Phylogeny, identification, and pathogenicity of *Lasiodiplodia* associated with postharvest stem-end rot of coconut in Brazil. Plant Dis. 100, 561–568. doi: 10.1094/PDIS-03-15-0242-RE, PMID: 30688600

[ref199] Ruiz-Aracil M. C. Valverde J. M. Ilea M. I. M. Valero D. Castillo S. Guillén F. (2024). Innovative postharvest management for Hass Avocado at the Preclimacteric stage: a combined technology with GABA and 1-MCP. Foods 13:2485. doi: 10.3390/foods13162485, PMID: 39200412 PMC11354002

[ref200] Salotti I. Ji T. Rossi V. (2022). Temperature requirements of *Colletotrichum* spp. belonging to different clades. Front. Plant Sci. 13:953760. doi: 10.3389/fpls.2022.953760, PMID: 35937340 PMC9354546

[ref201] Salvatore M. M. Giambra S. Naviglio D. DellaGreca M. Salvatore F. Burruano S.. (2018). Fatty acids produced by *Neofusicoccum vitifusiforme* and *N. parvum*, fungi associated with grapevine botryosphaeria dieback. Agriculture 8:189. doi: 10.3390/agriculture8120189

[ref202] Schaffer B. A. Wolstenholme B. N. Whiley A. W. (2013). The avocado: botany, production and uses. Wallingford, UK: CABI.

[ref203] Seethapathy P. Gurudevan T. Subramanian K. S. Kuppusamy P. (2016). Bacterial antagonists and hexanal-induced systemic resistance of mango fruits against *Lasiodiplodia theobromae* causing stem-end rot. J. Plant Interact. 11, 158–166. doi: 10.1080/17429145.2016.1252068

[ref204] Sellamuthu P. S. Mafune M. Sivakumar D. Soundy P. (2013). Thyme oil vapour and modified atmosphere packaging reduce anthracnose incidence and maintain fruit quality in avocado. J. Sci. Food Agric. 93, 3024–3031. doi: 10.1002/jsfa.6135, PMID: 23512681

[ref205] Serrano-García I. Domínguez-García J. Hurtado-Fernández E. González-Fernández J. J. Hormaza J. I. Beiro-Valenzuela M. G.. (2023). Assessing the RP-LC-MS-based metabolic profile of Hass avocados marketed in Europe from different geographical origins (Peru, Chile, and Spain) over the whole season. Plants 12:3004. doi: 10.3390/plants12163004, PMID: 37631215 PMC10458757

[ref206] Sharma G. Maymon M. Freeman S. (2017). Epidemiology, pathology and identification of *Colletotrichum* including a novel species associated with avocado (*Persea americana*) anthracnose in Israel. Sci. Rep. 7:15839. doi: 10.1038/s41598-017-15946-w, PMID: 29158592 PMC5696532

[ref207] Shezi S. Magwaza L. S. Tesfay S. Z. Mditshwa A. (2020). Biochemical changes in response to canopy position of avocado fruit (*cv*. ‘Carmen’ and ‘Hass’) during growth and development and relationship with maturity. Sci. Hortic. 265:109227. doi: 10.1016/j.scienta.2020.109227

[ref208] Shimshoni J. A. Bommuraj V. Chen Y. Sperling R. Barel S. Feygenberg O.. (2020). Postharvest fungicide for avocado fruits: antifungal efficacy and peel to pulp distribution kinetics. Foods 9:124. doi: 10.3390/foods9020124, PMID: 31979404 PMC7074524

[ref209] Shivachi B. Okoth M. Gikonge D. (2023). Status of avocado production, postharvest handling and utilization in Kenya: A review. East African J. Sci. Technol. Innov. 4. doi: 10.37425/eajsti.v4i.735

[ref210] Siddiqui Y. Ali A. (2014). “*Colletotrichum gloeosporioides* (anthracnose)” in Postharvest decay. ed. Bautista-Baños S. (San Diego: Academic Press), 337–371.

[ref211] Silva-Rojas H. V. Ávila-Quezada G. D. (2011). Phylogenetic and morphological identification of *Colletotrichum boninense*: a novel causal agent of anthracnose in avocado. Plant Pathol. 60, 899–908. doi: 10.1111/j.1365-3059.2011.02452.x

[ref212] Singh A. Singh V. K. Dwivedy A. K. Deepika Tiwari S. Dwivedi A.. (2020). “Biological control of plant diseases: opportunities and limitations” in Plant microbiome paradigm. eds. Varma A. Tripathi S. Prasad R. (Cham: Springer International Publishing), 121–146.

[ref213] Sivakumar D. Tuna Gunes N. Romanazzi G. (2021). A comprehensive review on the impact of edible coatings, essential oils, and their Nano formulations on postharvest decay anthracnose of avocados, mangoes, and papayas. Front. Microbiol. 12:711092. doi: 10.3389/fmicb.2021.711092 34394060 PMC8360855

[ref214] Slippers B. Crous P. Denman S. Coutinho T. Wingfield B. Wingfield M. (2004). Combined multiple gene genealogies and phenotypic characters differentiate several species previously identified as *Botryosphaeria dothidea*. Mycologia 96, 83–101. doi: 10.2307/3761991, PMID: 21148832

[ref215] Slippers B. Crous P. W. Jami F. Groenewald J. Z. Wingfield M. J. (2017). Diversity in the *Botryosphaeriales*: looking back, looking forward. Fungal Biol. 121, 307–321. doi: 10.1016/j.funbio.2017.02.002, PMID: 28317537

[ref216] Soares M. G. O. Alves E. Silveira A. L. Pereira F. D. Guimarães S. S. C. (2021). *Colletotrichum siamense* is the main aetiological agent of anthracnose of avocado in South-Eastern Brazil. Plant Pathol. 70, 154–166. doi: 10.1111/ppa.13262

[ref217] Sonavane P. S. Venkataravanappa V. (2022). “Avocado (*Persea americana* mill.) diseases and their management” in Diseases of horticultural crops: diagnosis and management (New York: Apple Academic Press).

[ref218] Stensvand A. Børve J. Talgø V. (2017). Overwintering diseased plant parts and newly infected flowers and fruit as sources of inoculum for *Colletotrichum acutatum* in sour cherry. Plant Dis. 101, 1207–1213. doi: 10.1094/PDIS-11-16-1599-RE, PMID: 30682962

[ref219] Stephenson S.-A. Green J. R. Manners J. M. Maclean D. J. (1997). Cloning and characterisation of glutamine synthetase from *Colletotrichum gloeosporioides* and demonstration of elevated expression during pathogenesis on *Stylosanthes Guianensis*. Curr. Genet. 31, 447–454. doi: 10.1007/s002940050228, PMID: 9162117

[ref220] Sun Y. Shuai L. Luo D. Ba L. (2023). The inhibitory mechanism of eugenol on *Lasiodiplodia theobromae* and its induced disease resistance of passion fruit. Agronomy 13:1408. doi: 10.3390/agronomy13051408

[ref221] Swart S. H. Serfontein J. J. Swart G. Labuschagne C. (2009). Chemical control of post-harvest diseases of mango: the effect of fludioxonil and prochloraz on soft brown rot, stem-end rot and anthracnose. Acta Hortic. 820, 503–510. doi: 10.17660/ActaHortic.2009.820.64

[ref222] Talhinhas P. Baroncelli R. (2021). *Colletotrichum* species and complexes: geographic distribution, host range and conservation status. Fungal Divers. 110, 109–198. doi: 10.1007/s13225-021-00491-9

[ref223] Tan G. H. Ali A. Yasmeen S. (2024). Volatile profiling of papaya fruit as an early detection tool for stem-end rot disease caused by *Lasiodiplodia theobromae*. Acta Hortic. 1396, 73–80. doi: 10.17660/ActaHortic.2024.1396.11

[ref224] Templeton M. D. Rikkerink E. H. A. Solon S. L. Crowhurst R. N. (1992). Cloning and molecular characterization of the glyceraldehyde-3-phosphate dehydrogenase-encoding gene and cDNA from the plant pathogenic fungus *Glomerella Cingulata*. Gene 122, 225–230. doi: 10.1016/0378-1119(92)90055-T, PMID: 1452034

[ref225] Tesfay S. Z. Mditshwa A. Magwaza L. S. (2021). Evaluation of carboxymethyl cellulose edible coating enriched with Moringa leaf and seed extracts’ performance on maintaining quality and shelf-life of avocado fruit. Acta Hortic. 1306, 335–340. doi: 10.17660/ActaHortic.2021.1306.43

[ref9013] Thilini Chethana K. W. Peng J. Li X. Xing Q. Liu M. Zhang W.. (2020). LtEPG1, a Secretory Endopolygalacturonase Protein, Regulates the Virulence of Lasiodiplodia theobromae in Vitis vinifera and Is Recognized as a Microbe-Associated Molecular Patterns. Phytopathology 110, 1727–1736. doi: 10.1094/PHYTO-04-20-0118-R 32460690

[ref226] Thorp T. G. Hutching D. Lowe T. Marsh K. B. (1997). Survey of fruit mineral concentrations and postharvest quality of New Zealand-grown ‘Hass’ avocado (*Persea americana* mill.). N. Z. J. Crop. Hortic. Sci. 25, 251–260. doi: 10.1080/01140671.1997.9514014

[ref227] Tian Q. Lu C. Wang S. Xiong Q. Zhang H. Wang Y.. (2017). Rapid diagnosis of soybean anthracnose caused by *Colletotrichum truncatum* using a loop-mediated isothermal amplification (LAMP) assay. Eur. J. Plant Pathol. 148, 785–793. doi: 10.1007/s10658-016-1132-2

[ref228] Tingwa P. Young R. (1974). The effect of calcium on the ripening of avocado (*Persea americana* mill.) fruits. J. Am. Soc. Hortic. Sci. 99, 540–542.

[ref229] Torres C. Camps R. Aguirre R. Besoain X. (2016). First report of *Diaporthe rudis* in Chile causing stem-end rot on ‘Hass’ avocado fruit imported from California. Plant Dis. 100:1951. doi: 10.1094/PDIS-12-15-1495-PDN

[ref230] Twizeyimana M. Förster H. McDonald V. Wang D. H. Adaskaveg J. E. Eskalen A. (2013a). Identification and pathogenicity of fungal pathogens associated with stem-end rot of avocado in California. Plant Dis. 97, 1580–1584. doi: 10.1094/PDIS-03-13-0230-RE, PMID: 30716830

[ref231] Twizeyimana M. McDonald V. Mayorquin J. S. Wang D. H. Na F. Akgül D. S.. (2013b). Effect of fungicide application on the management of avocado branch canker (formerly Dothiorella canker) in California. Plant Dis. 97, 897–902. doi: 10.1094/PDIS-06-12-0518-RE, PMID: 30722531

[ref233] Valencia A. L. Gil P. M. Latorre B. A. Rosales I. M. (2019). Characterization and pathogenicity of Botryosphaeriaceae species obtained from avocado trees with branch canker and dieback and from avocado fruit with stem end rot in Chile. Plant Dis. 103, 996–1005. doi: 10.1094/PDIS-07-18-1131-RE, PMID: 30840843

[ref232] Valencia Bernal V. Pegahrad Z. Kamali Dashtarzhaneh M. Khodadadi F. (2025). Identification, characterization, and fungicide sensitivity of Botryosphaeriaceae Fungi associated with avocado branch canker disease in Southern California. Plant Dis. doi: 10.1094/PDIS-12-24-2674-RE, PMID: 39946273

[ref9011] Valiente L. D. Parco K. M. R. Sangalang G. C. P. (2021). Non-destructive Image Processing Analysis for Defect Identification and Maturity Detection on Avocado Fruit. In 2021 5th International Conference on Communication and Information Systems (ICCIS). 175–179. doi: 10.1109/ICCIS53528.2021.9645970

[ref234] Vázquez-González Y. Prieto C. Lagaron J. M. Ragazzo-Sánchez J. A. Calderón-Santoyo M. (2024). Solution blow spinning to produce antifungal nanofibers from pullulan, fucoPol, and cashew gum and its potential postharvest application on avocado fruit. ACS Food Sci. Technol. 4, 3170–3181. doi: 10.1021/acsfoodscitech.4c00794

[ref235] Velázquez-del Valle M. G. Campos-Martínez A. Flores-Moctezuma H. E. Suárez-Rodríguez R. Ramírez-Trujillo J. A. Hernández-Lauzardo A. N. (2016). First report of avocado anthracnose caused by *Colletotrichum karstii* in Mexico. Plant Dis. 100:534. doi: 10.1094/PDIS-03-15-0249-PDN

[ref236] Vieira W. A. d. S. Bezerra P. A. da Silva A. C. Veloso J. S. Câmara M. P. S. Doyle V. P. (2020). Optimal markers for the identification of *Colletotrichum* species. Mol. Phylogenet. Evol. 143:106694. doi: 10.1016/j.ympev.2019.106694, PMID: 31786239

[ref237] Vivekananthan R. Ravi M. Ramanathan A. Samiyappan R. (2004). Lytic enzymes induced by *Pseudomonas fluorescens* and other biocontrol organisms mediate defence against the anthracnose pathogen in mango. World J. Microbiol. Biotechnol. 20, 235–244. doi: 10.1023/B:WIBI.0000023826.30426.f5

[ref9012] Wang F. Zhang F. Chen M. Liu Z. Zhang Z. Fu J.. (2017). Comparative Transcriptomics Reveals Differential Gene Expression Related to Colletotrichum gloeosporioides Resistance in the Octoploid Strawberry. Front. Plant Sci. 8:779. doi: 10.3389/fpls.2017.00779 28555149 PMC5430163

[ref238] Wang L. Hou H. Zhou Z. Tu H. Yuan H. (2021). Identification and detection of *Botryosphaeria dothidea* from kiwifruit (*Actinidia chinensis*) in China. Plants 10:401. doi: 10.3390/plants10020401, PMID: 33672451 PMC7923295

[ref239] Wang N. Huo Y.-X. (2022). Using genome and transcriptome analysis to elucidate biosynthetic pathways. Curr. Opin. Biotechnol. 75:102708. doi: 10.1016/j.copbio.2022.102708, PMID: 35278747

[ref240] Wanjiku E. K. Waceke J. W. Mbaka J. N. (2021). Suppression of stem-end rot on avocado fruit using *Trichoderma* spp. in the central highlands of Kenya. Adv. Agric. 2021:e8867858. doi: 10.1155/2021/8867858

[ref241] Wanjiku E. K. Waceke J. W. Wanjala B. W. Mbaka J. N. (2020). Identification and pathogenicity of fungal pathogens associated with stem end rots of avocado fruits in Kenya. Int. J. Microbiol. 2020:e4063697. doi: 10.1155/2020/4063697, PMID: 32695175 PMC7368227

[ref242] Wedding B. B. Wright C. Grauf S. Gadek P. White R. D. (2019). The application of FT-NIRS for the detection of bruises and the prediction of rot susceptibility of ‘Hass’ avocado fruit. J. Sci. Food Agric. 99, 1880–1887. doi: 10.1002/jsfa.9383, PMID: 30264542

[ref243] Wedding B. B. Wright C. Grauf S. White R. D. (2012). “The application of near infrared spectroscopy for the assessment of avocado quality attributes” in Infrared spectroscopy - life and biomedical sciences. ed. Theophanides T. (London, UK: Intech Open).

[ref244] Weir B. S. Johnston P. R. Damm U. (2012). The *Colletotrichum gloeosporioides* species complex. Stud. Mycol. 73, 115–180. doi: 10.3114/sim0011, PMID: 23136459 PMC3458417

[ref246] White A. Woolf A. Hofman P. Arpaia M. L. (2009). The international avocado quality manual. Davis, CA: UC Davis - Postharvest Technology Center.

[ref245] White T. Bruns T. Lee S. Taylor J. Innis M. Gelfand D.. (1990). “Amplification and direct sequencing of fungal ribosomal RNA genes for phylogenetics” in PCR protocols: a guide to methods and applications. New York, 315–322.

[ref247] Wijeratnam S. W. Dharmatilaka Y. Weerasinghe D. (2008). Host specificity of Colletotrichum gloeosporioides and Botryodiplodia theobromae isolates from mango, papaya and Rambutan and their response to Trichoderma harzianum, in Conference on international research on food security, natural resource management and rural development.

[ref248] Wong Y.-P. Othman S. Lau Y.-L. Radu S. Chee H.-Y. (2018). Loop-mediated isothermal amplification (lamp): a versatile technique for detection of micro-organisms. J. Appl. Microbiol. 124, 626–643. doi: 10.1111/jam.13647, PMID: 29165905 PMC7167136

[ref249] World Population Review (2025). Avocado consumption by country. Available online at: https://worldpopulationreview.com/country-rankings/avocado-consumption-by-country

[ref251] Wu C.-J. Lin M.-C. Ni H.-F. (2023). *Colletotrichum* species causing anthracnose disease on avocado fruit in Taiwan. Eur. J. Plant Pathol. 165, 629–647. doi: 10.1007/s10658-022-02635-2

[ref250] Wu J. Y. Hu X. R. Zhang C. Q. (2019). Molecular detection of QoI resistance in *Colletotrichum gloeosporioides* causing strawberry anthracnose based on loop-mediated isothermal amplification assay. Plant Dis. 103, 1319–1325. doi: 10.1094/PDIS-09-18-1593-RE, PMID: 30998417

[ref252] Xoca-Orozco L.-Á. Cuellar-Torres E. A. González-Morales S. Gutiérrez-Martínez P. López-García U. Herrera-Estrella L.. (2017). Transcriptomic analysis of avocado Hass (*Persea americana* mill) in the interaction system fruit-chitosan-*Colletotrichum*. Front. Plant Sci. 8:956. doi: 10.3389/fpls.2017.00956, PMID: 28642771 PMC5462954

[ref253] Xu C. Zhang H. Chi F. Ji Z. Dong Q. Cao K.. (2016). Species-specific PCR-based assays for identification and detection of Botryosphaeriaceae species causing stem blight on blueberry in China. J. Integr. Agric. 15, 573–579. doi: 10.1016/S2095-3119(15)61177-7

[ref255] Yang J. Duan K. Liu Y. Song L. Gao Q.-H. (2022). Method to detect and quantify colonization of anthracnose causal agent *Colletotrichum gloeosporioides* species complex in strawberry by real-time PCR. J. Phytopathol. 170, 326–336. doi: 10.1111/jph.13082

[ref254] Yan J. Y. Zhao W. S. Chen Z. Xing Q. K. Zhang W. Chethana K. W. T.. (2018). Comparative genome and transcriptome analyses reveal adaptations to opportunistic infections in woody plant degrading pathogens of Botryosphaeriaceae. DNA Res. 25, 87–102. doi: 10.1093/dnares/dsx040, PMID: 29036669 PMC5824938

[ref256] Yoshida S. Tsukiboshi T. Shinohara H. Koitabashi M. Tsushima S. (2007). Occurrence and development of *Colletotrichum acutatum* on symptomless blueberry bushes. Plant Pathol. 56, 871–877. doi: 10.1111/j.1365-3059.2007.01645.x

[ref257] Younis I. Y. Khattab A. R. Selim N. M. Sobeh M. Elhawary S. S. Bishbishy M. H. E. (2022). Metabolomics-based profiling of 4 avocado varieties using HPLC–MS/MS and GC/MS and evaluation of their antidiabetic activity. Sci. Rep. 12:4966. doi: 10.1038/s41598-022-08479-4, PMID: 35322072 PMC8943142

[ref258] Zahid N. Maqbool M. Siddiqui Y. Manickam S. Ali A. (2015). Regulation of inducible enzymes and suppression of anthracnose using submicron chitosan dispersions. Sci. Hortic. 193, 381–388. doi: 10.1016/j.scienta.2015.07.014

[ref259] Zamir D. Galsurker O. Alkan N. Eltzov E. (2020). Detection of quiescent fungi in harvested fruit using CMOS bosensor: a proof of concept study. Talanta 217:120994. doi: 10.1016/j.talanta.2020.120994 32498883

[ref260] Zhang B. Gao X. Wang Q. Li Y. He C. Luo H.. (2022). Integrated application of transcriptomics and metabolomics provides insights into the antifungal activity of Α-Phenylcinnamic acid against *Colletotrichum gloeosporioides*. Postharvest Biol. Technol. 186:111834. doi: 10.1016/j.postharvbio.2022.111834

[ref9014] Zhang W. Yan J. Li X. Xing Q. Chethana K. W. T. Zhao W. (2019). Transcriptional response of grapevine to infection with the fungal pathogen *Lasiodiplodia theobromae*. Sci Rep. 9:5387. doi: 10.1038/s41598-019-41796-9 30926851 PMC6441073

